# Identification of Dipeptidyl Peptidase (DPP) Family Genes in Clinical Breast Cancer Patients via an Integrated Bioinformatics Approach

**DOI:** 10.3390/diagnostics11071204

**Published:** 2021-07-02

**Authors:** Tak-Kee Choy, Chih-Yang Wang, Nam Nhut Phan, Hoang Dang Khoa Ta, Gangga Anuraga, Yen-Hsi Liu, Yung-Fu Wu, Kuen-Haur Lee, Jian-Ying Chuang, Tzu-Jen Kao

**Affiliations:** 1 Department of Surgery, Division of Gastroenterologic Surgery, Yuan’s General Hospital, Kaohsiung 80249, Taiwan; ahkai_090@kimo.com; 2 PhD Program for Cancer Molecular Biology and Drug Discovery, College of Medical Science and Technology, Taipei Medical University, Taipei 11031, Taiwan; chihyang@tmu.edu.tw (C.-Y.W.); d621109004@tmu.edu.tw (H.D.K.T.); g.anuraga@unipasby.ac.id (G.A.); khlee@tmu.edu.tw (K.-H.L.); 3 Graduate Institute of Cancer Biology and Drug Discovery, College of Medical Science and Technology, Taipei Medical University, Taipei 11031, Taiwan; 4 NTT Institute of Hi-Technology, Nguyen Tat Thanh University, Ho Chi Minh City 700000, Vietnam; pnnam@ntt.edu.vn; 5 Department of Statistics, Faculty of Science and Technology, PGRI Adi Buana University, East Java, Surabaya 60234, Indonesia; 6 School of Chinese Medicine for Post-Baccalaureate, I-Shou University, Kaohsiung 82445, Taiwan; isu10556037a@cloud.isu.edu.tw; 7 National Defense Medical Center, Department of Medical Research, School of Medicine, Tri-Service General Hospital, Taipei 11490, Taiwan; qrince@yahoo.com.tw; 8 Cancer Center, Wan Fang Hospital, Taipei Medical University, Taipei 11031, Taiwan; 9 Graduate Institute of Neural Regenerative Medicine, College of Medical Science and Technology, Taipei Medical University, Taipei 11031, Taiwan; chuangcy@tmu.edu.tw; 10 Research Center of Neuroscience, Taipei Medical University, Taipei 11031, Taiwan; 11 TMU Research Center of Cancer Translational Medicine, Taipei Medical University, Taipei 11031, Taiwan

**Keywords:** DPP family genes, breast cancer, bioinformatics

## Abstract

Breast cancer is a heterogeneous disease involving complex interactions of biological processes; thus, it is important to develop therapeutic biomarkers for treatment. Members of the dipeptidyl peptidase (DPP) family are metalloproteases that specifically cleave dipeptides. This family comprises seven members, including DPP3, DPP4, DPP6, DPP7, DPP8, DPP9, and DPP10; however, information on the involvement of DPPs in breast cancer is lacking in the literature. As such, we aimed to study their roles in this cancerous disease using publicly available databases such as cBioportal, Oncomine, and Kaplan–Meier Plotter. These databases comprise comprehensive high-throughput transcriptomic profiles of breast cancer across multiple datasets. Furthermore, together with investigating the messenger RNA expression levels of these genes, we also aimed to correlate these expression levels with breast cancer patient survival. The results showed that DPP3 and DPP9 had significantly high expression profiles in breast cancer tissues relative to normal breast tissues. High expression levels of DPP3 and DPP4 were associated with poor survival of breast cancer patients, whereas high expression levels of DPP6, DPP7, DPP8, and DPP9 were associated with good prognoses. Additionally, positive correlations were also revealed of DPP family genes with the cell cycle, transforming growth factor (TGF)-beta, kappa-type opioid receptor, and immune response signaling, such as interleukin (IL)-4, IL6, IL-17, tumor necrosis factor (TNF), and interferon (IFN)-alpha/beta. Collectively, DPP family members, especially DPP3, may serve as essential prognostic biomarkers in breast cancer.

## 1. Introduction

Approximately 30% of all cancers that occurred in women in the United States in 2020 were breast cancer [1]. Breast cancer is subtyped by the expression levels of the estrogen receptor (ER, the gene of which is named *ESR1*), progesterone receptor (PR), and human epidermal growth factor receptor (HER)-2. Many genetic therapies are applied to breast cancer patients, such as fulvestrant [2,3], cyclin-dependent kinase inhibitors [4], aromatase-related inhibitors [5], and histone deacetylase (HDAC) inhibitors [6]. It has been reported that 70% of metastatic breast cancer cases have high expression of B-cell lymphoma 2 (BCL2). Using BCL2 inhibitors for these metastatic cases improved cancer cell apoptosis in a preclinical model of breast cancer [7,8]. Genes that are involved in this biological process are dipeptidyl peptidase (*DPP*) family genes, extracellular-signal-regulated kinase (*ERK*), GATA-binding protein 3 (*GATA3*), signal transducer and activator of transcription 3 (*STAT3*), phosphatidylinositol 3-kinase (*PI3K*), and *NOTCH* [9,10,11,12].

Members of the DPP family are metalloproteases that specifically cleave dipeptides, and this family is comprised of seven members, including DPP3, DPP4, DPP6, DPP7, DPP8, DPP9, and DPP10, which are zinc-dependent hydrolases involved in degrading oligopeptides. Many biological processes involve these proteins, including cancer cell defense against oxidative stress. A previous study demonstrated that DPP3 and DPP7 are highly expressed in multiple myelomas [13]. DPP3 overexpression was positively associated with KEAP1 mutant tumors, and it further promoted lung cancer development [14]. DPP4 attenuates *C*-X-*C* motif ligand 10 (CXCL10) and atypical chemokine receptor 2 (ACKR2) activity by regulating N-terminal processing [15], while DPP4 inhibitors may serve as second-line treatment for epithelial ovarian cancer [16]. High expression levels of DPP4 in some types of cancer patients can increase susceptibility to severe acute respiratory syndrome coronavirus (SARS-CoV)-2 infection and further cause cytokine storms [17]. DPP6 promoter activity was significantly higher in pancreatic ductal adenocarcinoma tissues compared to normal tissues [18]. Knockdown of DPP7 increased apoptosis by upregulating Bax–Bcl2 signaling in the HepG2 liver cancer cell line [19]. NLR family pyrin domain-containing 1 (NLRP1) can interact with DPP8 and DPP9, which can serve as a checkpoint for activating the NLRP1 inflammasome [20]. A DPP8 and DPP9 inhibitor can promote apoptosis by activating poly(ADP ribose) polymerase (PARP) and caspase-3 in multiple myelomas [21]. DPP10 inhibits colon cancer stem cell proliferation by regulating microRNAs such as miR-127-3p [22].

Although correlations between breast cancer and DPP family members′ messenger (m)RNA expression levels still remain unclear, it is important to investigate this correlation with a comprehensive, holistic approach. It is well known that high-throughput technologies provide thousands to millions of data points from a single run, making them highly suitable tools for rapidly and efficiently screening potential biomarkers [23,24,25]. Significant alterations in transcriptomic levels of genes imply their roles in a certain disease, such as oncogenic or tumor suppressors in cancerous diseases [26]. Utilizing this concept, we queried publicly available transcriptomic databases for DPP mRNA expression levels in many breast cancer datasets, including multiple breast cancer subtypes. Furthermore, protein and gene interaction networks were evaluated to screen for downstream molecules associated with DPP family member genes.

## 2. Materials and Methods

### 2.1. Oncomine and GEPIA Analyses

To search for mRNA expression levels of DPP genes in 20 types of common cancers relative to normal matched tissue, we used Oncomine (www.oncomine.org, accessed on 01 May 2021) and GEPIA (http://gepia.cancer-pku.cn/, accessed on 01 May 2021) [27,28,29,30,31,32]. Search thresholds included the multiple of change (>2.0), *p* value (<10^−4^), and gene ranking percentile (top 10%). Search results displayed the number of datasets qualified for the above thresholds with up- and downregulated expression levels in different types and subtypes of cancers. Red- and blue-colored gradients were used to show these genes′ up- and downregulated expression levels based on the top-ranking percentiles.

### 2.2. Cancer Cell Line Encyclopedia (CCLE) Analysis

Additionally, we used the CCLE database (https://portals.broadinstitute.org/ccle, accessed on 1 May 2021) to search for expression levels of *DPP* genes in cancer cell lines [33]. The CCLE is comprised of many human cancer cell lines (*n* = 1457) with large numbers of unique datasets (*n* = 136,488). Gene expression levels were retrieved using an RNA sequencing method in 60 breast cancer cell lines and the data were plotted with default settings as we previously described [34,35,36,37].

### 2.3. Kaplan–Meier (KM) Plot of Survival Analysis

Gene expression levels correlations of mRNAs of *DPP* genes with breast cancer patients’ survival, such as relapse-free survival (RFS), were investigated using the KM plotter database (https://kmplot.com/, accessed on 1 May 2021) [38]. The breast cancer database was established using gene expression data and survival information of 2898 patients acquired from the Gene Expression Omnibus (GEO) (Affymetrix HGU133A microarrays platform). The numbers of patients in high- and low-risk groups were also displayed along with the survival duration on the horizontal axis. Poor survival status of patients was based on log-rank *p* values smaller than 0.05 for statistically significant differences between low and high mRNA expression of the target genes. The HR ratio was displayed as a mean, together with 95% confidence intervals (CI). All analyses in the KM plotter database were performed with default parameters for calculating survival curves, log-rank *p* values, as well as hazard ratios (HRs) with 95% CIs.

### 2.4. Functional Enrichment Analysis of DPP Family Members

To obtain shared coexpressed genes with *DPP* genes between The Cancer Genome Atlas (TCGA) and Metabric from Cbioportal databases, the final top 10% of coexpressed genes were further uploaded into MetaCore software (https://portal.genego.com/, accessed on 1 May 2021) for pathway and network analyses with Gene Ontology (GO). A log-rank *p* value of <0.05 was considered to be statistically significant [39,40,41].

### 2.5. Statistical Analysis

The Cox proportional hazard model was also utilized to evaluate the role of clinicopathological features in overall survival (OS) results from the TCGA database. Patients were differentiated into low- and high-expression groups by applying a median cutoff strategy. Extracted clinical data for patients were managed using R software using “survival” and “survminer”. Cox univariate and multivariate analyses were separately performed to construct a proportional hazard model. Data were obtained from TCGA Pancancer Atlas and clinical data for patients were extracted and managed with R language. Comparisons between groups were done using Student’s *t*-test. The mRNA expression level was transformed into logarithmic scale (log_2_(TPM + 1)). The *q* value was set to 0.05 for GEPIA analysis. A *p* value of <0.05 was used to make statistically significant decisions, as previously described [42,43].

## 3. Results

### 3.1. DPP Family Members Play Crucial Roles in Breast Cancer Development

**Figure 1:**
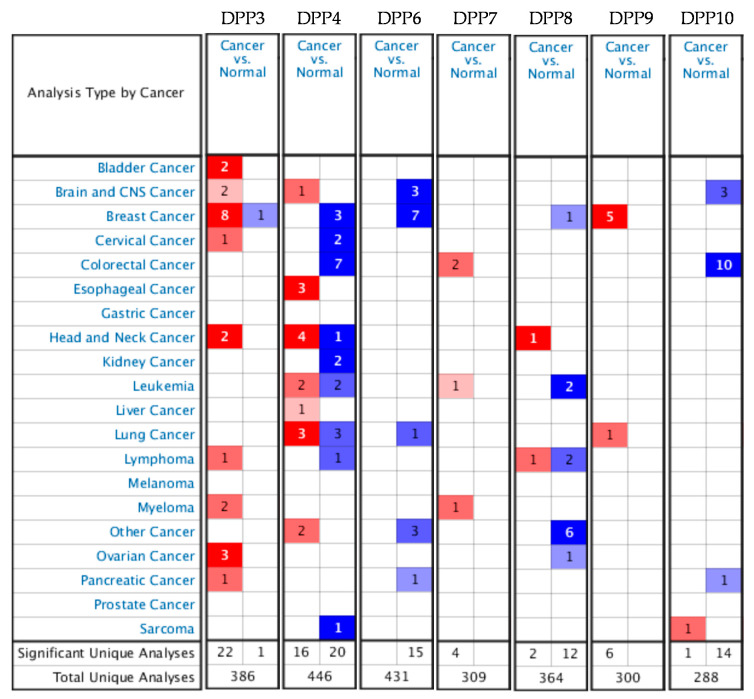
The mRNA expression levels of dipeptidyl peptidase (DPP) family genes in pan-cancers from the Oncomine database. The analysis was done on mRNA expression levels of breast cancer tissues and normal matched tissues. Red- and blue-colored gradients show gene rank percentiles in specific datasets. The significant unique analysis represents the number of datasets that reached the threshold over total unique analyses.

Previous studies identified seven DPP family members in humans; some of their members were reported to be crucially involved in cancer development. Consequently, a meta-analysis study of the roles of these genes is necessary to clarify their roles in breast cancer and its subtypes, which might provide potential biomarkers for this disease. Results from an Oncomine analysis showed that mRNA expression levels of DPP3 and DPP9 were highly upregulated in breast cancer tissues, whereas DPP4, DPP6, and DPP8 exhibited downregulated levels in breast cancer tissues relative to normal breast tissues (Figure 1).

### 3.2. Associations of DPP Family Members with Clinicopathological Parameters in Breast Cancer

**Figure 2:**
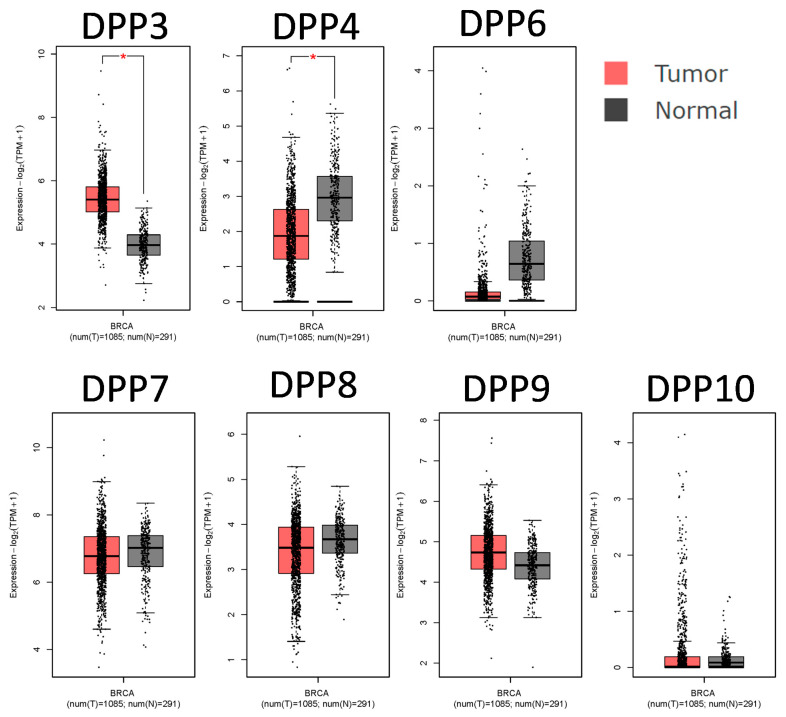
Transcript expression levels of dipeptidyl peptidase (*DPP*) family genes in breast cancers. The red box plots show tumor expression levels, while gray represents normal breast tissues.

**Figure 3:**
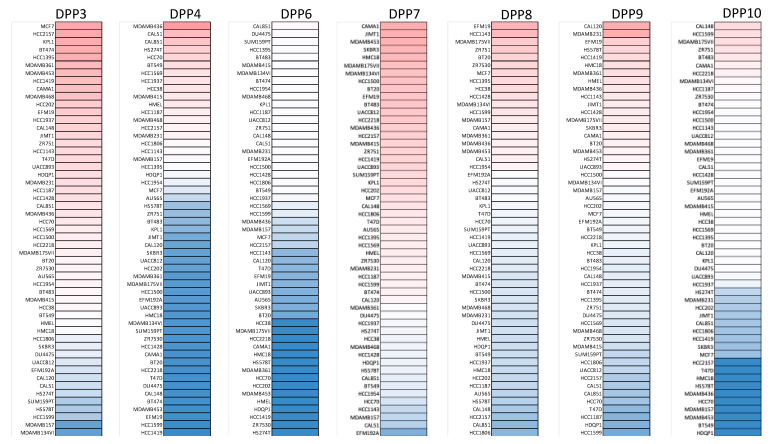
Transcript expression levels of dipeptidyl peptidase (*DPP*) family genes in a variety of cancer cell lines. Differential expression levels of DPP3, DPP4, DPP6, DPP7, DPP8, DPP9, and DPP10 in the Cancer Cell Line Encyclopedia (CCLE). The upper blocks represented by red indicate overexpression, whereas the bottom blocks indicate underexpression.

The mRNA expression levels of *DPP* genes in breast cancer tissues and normal tissues were compared with the GEPIA tool. DPP3 and DPP9 mRNA expression levels were upregulated in breast cancer tissues relative to normal breast tissues (Figure 2) and other subtypes (Supplementary Figure S1). Additionally, the CCLE analysis also presented mRNA expression levels of DPP family members in breast cancer cell lines (Figure 3).

### 3.3. Genes Coexpressed with DPP Family Members in Breast Cancer

**Figure 4:**
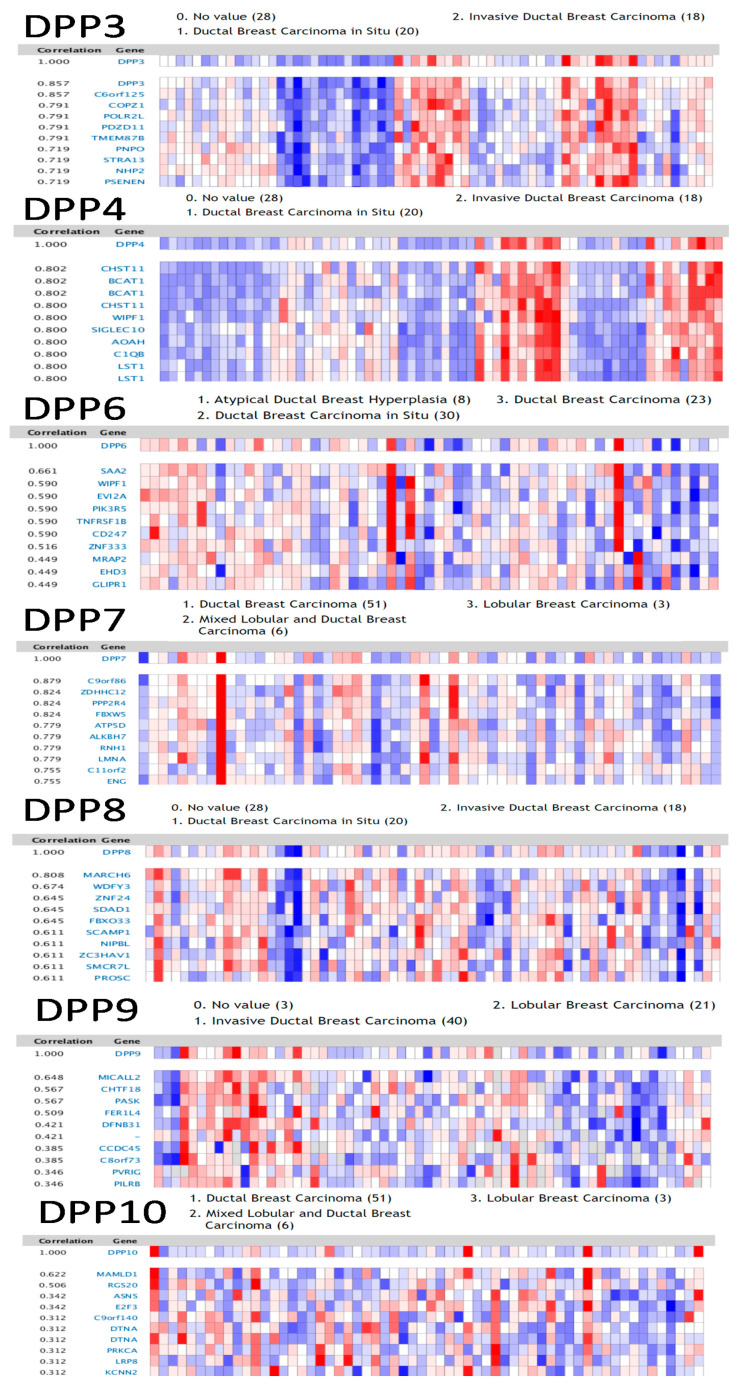
Genes coexpressed with dipeptidyl peptidase (DPP) family members and correlations between DPP family genes in breast cancer patients. Genes coexpressed with the DPP3, DPP4, DPP6, DPP7, DPP8, DPP9, and DPP10 genes in breast cancer patients from the Oncomine platform are presented in a heatmap format.

Gene coexpression levels with DPP3 in the Ma dataset was analyzed via the Oncomine platform. We found that DPP3 was positively correlated with *C6orf125*, *COPZ1*, *POLRZL*, *PDZD11*, *TMEM87B*, *PNPO*, *STRA13*, *NHP2*, *PSENEN*, *CANT1*, *FKBP4*, *CYB561*, *PRR15L*, *MAPK13*, *GTF2IRD1*, *NR2F6*, and *KRT18*. We found that DPP4 expression was positively correlated with *CHST11*, *BCAT1*, *CHST11*, *WIPF1*, *SIGLEC10*, *AOAH*, *C1QB*, *LST1*, *S100A4*, *HLA-DOA*, *PLEKHOZ*, *SELPLG*, *FCGRZA*, *FCGR2A*, *FCGR3B*, *MSR1*, *ADAP2*, and *LAPTM5*. We found that DPP6 expression was positively correlated with *SAA2*, *WIPF1*, *EVIZA*, *PIK3R5*, *TNFRSFIB*, *CD247*, *ZNF333*, *MRAP2*, *EHD3*, *GLIPR1*, *FIBIN*, *SNCAIP*, *CMTM3*, *Cl0orf54*, *ID4*, *ABCB1*, *LPAR6*, *OGFRL1*, and *DAB2*. We found that DPP7 expression was positively correlated with *C9orf86*, *ZDHHC12*, *PPP2R4*, *FBXW5*, *ATP5D*, *ALKBH7*, *RNH1*, *LMNA*, *Cllorf2*, *ENG*, *MACROD1*, *ASL*, *PACS2*, *KIAA0562*, *SCOZ*, *ZNHIT2*, *PTOVI*, *NME4*, *ZMAT5*, and *CLDN4*. We found that DPP8 expression was positively correlated with *MARCH6*, *WDFY3*, *ZNF24*, *SDAD1*, *FBX033*, *SCAMP1*, *NIPBL*, *ZC3HAVI*, *SMCR7L*, *PROSC*, *RNF160*, *SRP72*, *G2E3*, *RNF13*, *CNOT7*, *ZNF148*, *VPS24*, *EIF5*, and *C9orf5*. We found that DPP9 expression was positively correlated with *SLC43A2*, *LOC338799*, *C16orf53*, *MICALL2*, *CHTF18*, *PASK*, *FERIL4*, *DFNB31*, *CCDC45*, *C8orf73*, *PVRIG*, *PILRB*, *LRCH4*, and *YPEL3*. We found that DPP10 expression was positively correlated with *MAMLD1*, *RGS20*, *ASNS*, *E2F3*, *C9orf140*, *DTNA*, *PRKCA*, *LRP8*, *KCNN2*, *TMSB15A*, *LOC286052*, *TMEM65*, *CHD7*, *GGH*, C2lorf30, *ANKS6*, *CLTCL1*, *EIF5A2*, and *HS3ST3Al* (Figure 4).

### 3.4. Protein Expression Levels and Prognostic Values of DPP Family Members in Breast Cancer

**Figure 5:**
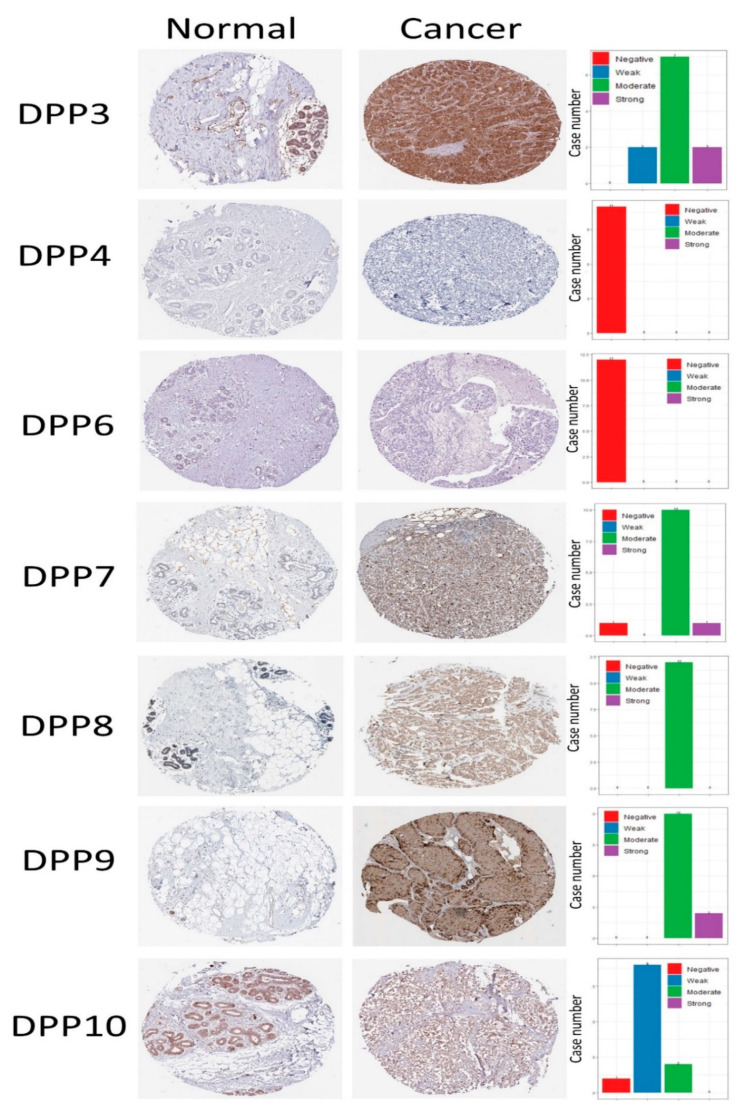
Protein expression levels of dipeptidyl peptidase (DPP) family members across clinical breast cancer specimens from the Human Protein Atlas. DPP3, DPP7, DPP8, DPP9, and DPP10 showed medium protein expression levels, while some clinical tissues showed strong positive expression levels of DPP3, DPP7, and DPP9 in breast cancer specimens.

**Figure 6:**
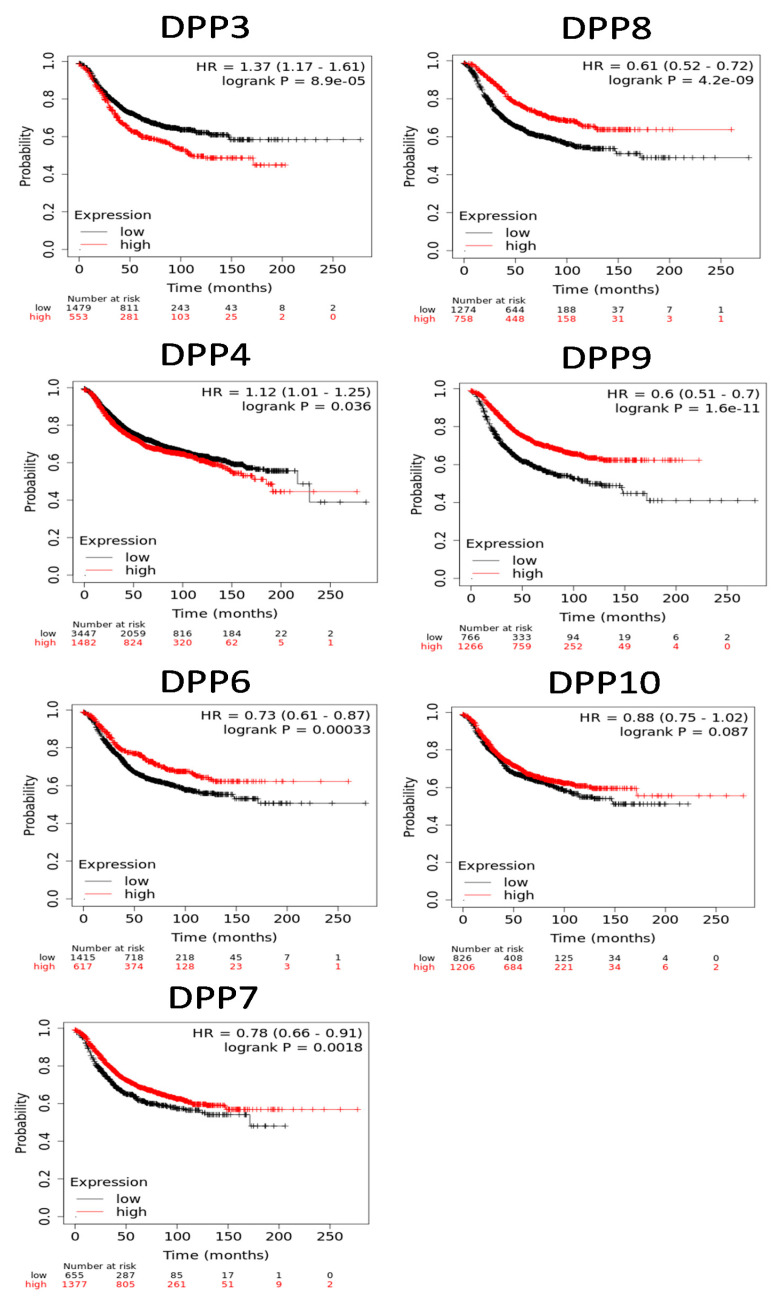
Relationships of expression levels of dipeptidyl peptidase (DPP) family members and recurrence-free survival (RFS) of clinical breast cancer patients (n = 2898). Kaplan–Meier plots show correlations of RFS with high and low expression levels of DPP family genes. Red and black lines indicate higher and lower values than the median, respectively. High expression levels of DPP3 and DPP4 were associated with poor survival, whereas high expression levels of DPP6, DPP7, DPP8, and DPP9 were associated with better survival rates (p < 0.05 considered significant).

After performing a screening of expression levels of DPP family members in breast cancer patients, we further explored the DDP members’ roles in clinical human breast cancer specimens in different molecular subtypes of breast cancer and their correlations with other featured biomarkers. To determine expression levels of DPP family members and their clinical relevance, the Human Protein Atlas (HPA) was used to analyze the protein expression levels of DPP family members in clinical specimens (https://www.proteinatlas.org/, accessed on 1 May 2021). Data demonstrated that DPP3, DPP7, DPP8, DPP9, and DPP10 mostly had medium protein expression levels, while some clinical tissues showed strong positive expression levels of DPP3, DPP7, and DPP9 in breast cancer specimens (Figure 5). The Kaplan–Meier (KM) plot showed that high expression levels of DPP3 and DPP4 were correlated with poor survival of breast cancer patients, whereas other DPP family members were not. These data implied DPP3 and DPP4′s oncogenic roles in breast cancer progression (Figure 6). In addition, multivariate analysis indicated that “treatment” and “tumor stage” were significantly associated with high-risk factors, while DPP3 expression levels were an independent survival determinant in breast cancer patients (Supplementary Figure S2).

### 3.5. Pathway and Network Analysis of DPP Family Member Genes

Enriched biological processes shown by the GeneGo Metacore analysis demonstrated that genes coexpressed with DPP family genes were involved in molecular processes related to cancer development. Furthermore, biological networks established by GeneGo Metacore from the pool of input genes also explained the biological processes associated with each tissue. Genes coxpressed with DPP family members from TCGA and METABRIC breast cancer datasets were uploaded to the MetaCore platform. Results showed that many cancer progression-related pathways were correlated with expression levels of *DPP* family genes. A strong cluster of the top 10% of coexpressed genes was obtained from TCGA and METABRIC breast cancer datasets. Next, GeneGo Metacore annotations of enriched biological processes revealed that genes coexpressed with DPP3 were involved in cell-cycle-related pathways and networks, such as “Cell cycle_Role of APC in cell cycle regulation”, “Cell cycle_Spindle assembly and chromosome separation” and “DNA dam-age_ATM/ATR regulation of G_2_/M checkpoint: cytoplasmic signaling” playing essential roles in breast cancer patients (Supplementary Figure S3, Table S1). Genes coexpressed with DPP4 were involved in cell TGF-related pathways and networks, such as “IL-1 beta- and Endothelin-1-induced fibroblast/myofibroblast migration and extracellular matrix production in asthmatic airways”, “Development_TGF-beta-dependent induction of EMT via SMADs”, “Expression targets of tissue factor signaling in cancer”, “Cell adhesion_ECM remodeling“, and “TGF-beta-induced fibroblast/myofibroblast migration and extracellular matrix production in asthmatic airways” playing essential roles in breast cancer patients (Supplementary Figure S4, Table S2). Genes coexpressed with DPP6 were involved in cell Kappa-type opioid receptor-related pathways and networks, such as “Muscle contraction_Role of kappa-type opioid receptor in heart”, “Development_Schema: FGF signaling in embryonic stem cell self-renewal and differentiation”, and “Neurophysiological process_Kappa-type opioid receptor signaling in the central nervous system” playing essential roles in breast cancer patients (Supplementary Figure S5, Table S3). Genes coexpressed with DPP7 were involved in cell cycle-related pathways and networks such as “Cell cycle_Role of SCF complex in cell cycle regulation”, “DNA damage_ATM/ATR regulation of G1/S checkpoint”, ”Cell cycle_Role of APC in cell cycle regulation“, ”Cell cycle_Spindle assembly and chromosome separation”, and “Cell cycle_Chromosome condensation in prometaphase“ playing essential roles in breast cancer patients (Supplementary Figure S6, Table S4). Genes coexpressed with DPP8 were involved in immune-related pathways and networks such as “IL-6 signaling in breast cancer cells”, ”G-protein signaling_Regulation of Cyclic AMP levels by ACM”, “Development_YAP/TAZ-mediated coregulation of transcription”, and “Immune response_IL-4-induced regulators of cell growth, survival, differentiation, and metabolism“, playing essential roles in breast cancer patients (Supplementary Figure S7, Table S5). Genes coexpressed with DPP9 involved immune-related pathways and networks, such as “Immune response_IFN-alpha/beta signaling via PI3K and NF-κB pathways”, “Immune response_TNF-R2 signaling pathways”, ”Development_GM-CSF signaling“, ”Main growth factor signaling cascades in multiple myeloma cells”, and “Apoptosis and survival_IL-17-induced CIKS-independent signaling pathways” playing essential roles in breast cancer patients (Supplementary Figure S8, Table S6). Genes coexpressed with DPP10 were involved cell cycle-related pathways and networks such as “Cell cycle_Role of APC in cell cycle regulation”, “Higher ESR1/ESR2 ratio in breast cancer”, “Cell cycle_The metaphase checkpoint”, ”Putative pathways of hormone action in neurofibromatosis type 1”, and “Cell cycle_Role of Nek in cell cycle regulation” playing essential roles in breast cancer patients (Supplementary Figure S9, Table S7).

## 4. Discussion

Breast cancer is the most-common cancer disease occurring in female subjects relative to other cancer types. Efforts and knowledge have improved over decades of study; however, treatment targets are still a focus of research for advanced stages and metastatic breast cancer. Consequently, developing and proposing new targets would benefit breast cancer patients [44]. Proteases widely participate in biological processes and regulate molecular functions, which can further promote cancer development. DPP3 was reported to regulate the genesis of leukemia and other malignancies [45]. This evidence was consistent with our data, as we found that DPP3 had high expression levels in breast cancer tissues at both the transcription and protein levels, and further caused poor prognoses in breast cancer patients. DPP4 was reported to be a therapeutic target for coronavirus pandemics, such as the Middle East respiratory syndrome coronavirus (MERS-CoV) and severe acute respiratory syndrome (SARS-CoV)-2 (i.e., coronavirus disease 2019 (COVID-19)) [46,47,48]. DPP4 contributes to ferroptosis in clear cell renal cell carcinoma [49], while DPP4 had high mRNA expression under hypoxic growth in ovarian cancer cells [50]. Interestingly, our data demonstrated that DPP4 had low expression levels in breast cancer tissues at both the transcription and protein levels, but was associated with poor prognoses in breast cancer patients. Therefore, DPP4 may be regulated by post-translational modifications (PTMs) or epigenetic-related mechanisms [51,52]. DPP6 served as a tumor-specific hypermethylated gene [53] and was significantly related to the prognosis of clear cell renal cell carcinoma patients [54]. Our data showed that DPP6 had low expression levels in breast cancer tissues at both the transcription and protein levels, and was further related to good prognoses in breast cancer patients, which also suggested that DPP6 may act as a tumor suppressor in cancer development. DPP7 had high expression levels in colorectal cancer patients and could be a significant predictor of a poor prognosis [55]; this is also consistent with our Oncomine and GEPIA analyses. We found that DDP7 was highly expressed in colon cancer tissues but not in breast cancer patients; therefore, the role of DPP7 in cancer progression may occur in a tissue-specific manner. DPP8 and DPP9 can regulate pyroptosis in human acute myeloid leukemia [56], while DPP8 and DPP9 mRNAs are overexpressed in ovarian carcinoma [57]. Our data showed that DPP8 had low expression levels in breast cancer tissues at both the transcription and protein levels whereas DPP9 did not, and both of them were related to good prognoses in breast cancer patients. DPP10 displayed significant correlations with methylation levels and cervical neoplasia progression [58]. DPP10 was underexpressed in primary glioblastomas [59], and was also found to be down-expressed in nasopharyngeal carcinoma [60]. These data are very similar to our analysis, as our data showed that DPP10 had low expression levels in breast cancer tissues and was further related to good prognoses in breast cancer patients. The literature on the roles of DPP family members in breast cancer is still limited; therefore, the present study can provide valuable information for prospective studies in breast cancer research.

## 5. Conclusions

In summary, the present study provides new findings related to DPP family genes, which have prognostic and predictive values in breast cancer, as validated by multiple datasets. Comprehensive analysis of DPP gene members in breast cancer could serve as novel biomarkers of breast cancer.

## Data Availability

Not applicable.

## Supplementary Materials

Supplementary materials can be found at https://www.mdpi.com/article/10.3390/diagnostics11071204/s1. Figure S1. Transcription levels of dipeptidyl peptidase (*DPP*) family genes in different subtypes of breast cancer patients (TCGA database). Figure S2. Multivariate analysis of dipeptidyl peptidase 3 (DPP3) expression and relationships between it and clinicopathological parameters (age, treatment, stage, and TNM (tumor, node, metastasis) stage). Figure S3. MetaCore pathway analysis of the coexpression gene network of dipeptidyl peptidase 3 (DPP3) in breast cancer patients. Figure S4. MetaCore pathway analysis of the coexpression gene network of dipeptidyl peptidase 4 (DPP4) in breast cancer patients. Figure S5. MetaCore pathway analysis of the coexpression gene network of dipeptidyl peptidase 6 (DPP6) in breast cancer patients. Figure S6. MetaCore pathway analysis of the coexpression gene network of dipeptidyl peptidase 7 (DPP7) in breast cancer patients. Figure S7. MetaCore pathway analysis of the coexpression gene network of dipeptidyl peptidase 8 (DPP8) in breast cancer patients. Figure S8. MetaCore pathway analysis of the coexpression gene network of dipeptidyl peptidase 9 (DPP9) in breast cancer patients. Figure S9. MetaCore pathway analysis of the coexpression gene network of dipeptidyl peptidase 10 (DPP10) in breast cancer patients. Table S1. Pathway analysis of dipeptidyl peptidase 3 (DPP3)-coexpressed genes from public breast cancer databases using the MetaCore database (with *p* < 0.01 set as the cut-off value). Table S2. Pathway analysis of dipeptidyl peptidase 4 (DPP4)-coexpressed genes from public breast cancer databases using the MetaCore database (with *p* < 0.01 set as the cut-off value). Table S3. Pathway analysis of dipeptidyl peptidase 6 (DPP6)-coexpressed genes from public breast cancer databases using the MetaCore database (with *p* < 0.01 set as the cut-off value). Table S4. Pathway analysis of dipeptidyl peptidase 7 (DPP7)-coexpressed genes from public breast cancer databases using the MetaCore database (with *p* < 0.01 set as the cut-off value). Table S5. Pathway analysis of dipeptidyl peptidase 8 (DPP8)-coexpressed genes from public breast cancer databases using the MetaCore database (with *p* < 0.01 set as the cut-off value). Table S6. Pathway analysis of dipeptidyl peptidase 9 (DPP9)-coexpressed genes from public breast cancer databases using the MetaCore database (with *p* < 0.01 set as the cut-off value). Table S7. Pathway analysis of dipeptidyl peptidase 10 (DPP10)-coexpressed genes from public breast cancer databases using the MetaCore database (with *p* < 0.01 set as the cut-off value).
