# Supplementary material for: Identification of Dipeptidyl Peptidase (DPP) Family Genes in Clinical Breast Cancer Patients via an Integrated Bioinformatics Approach
Source: Diagnostics (Basel). 2021 Jul 2;11(7):1204. doi: 10.3390/diagnostics11071204 (PMC8304478; doi:10.3390/diagnostics11071204)
Supplement: Supplementary file 1 [file diagnostics-11-01204-s001.zip › diagnostics-1256375-supplementary.pdf]

diagnostics

MDPI

Supplementary Materials

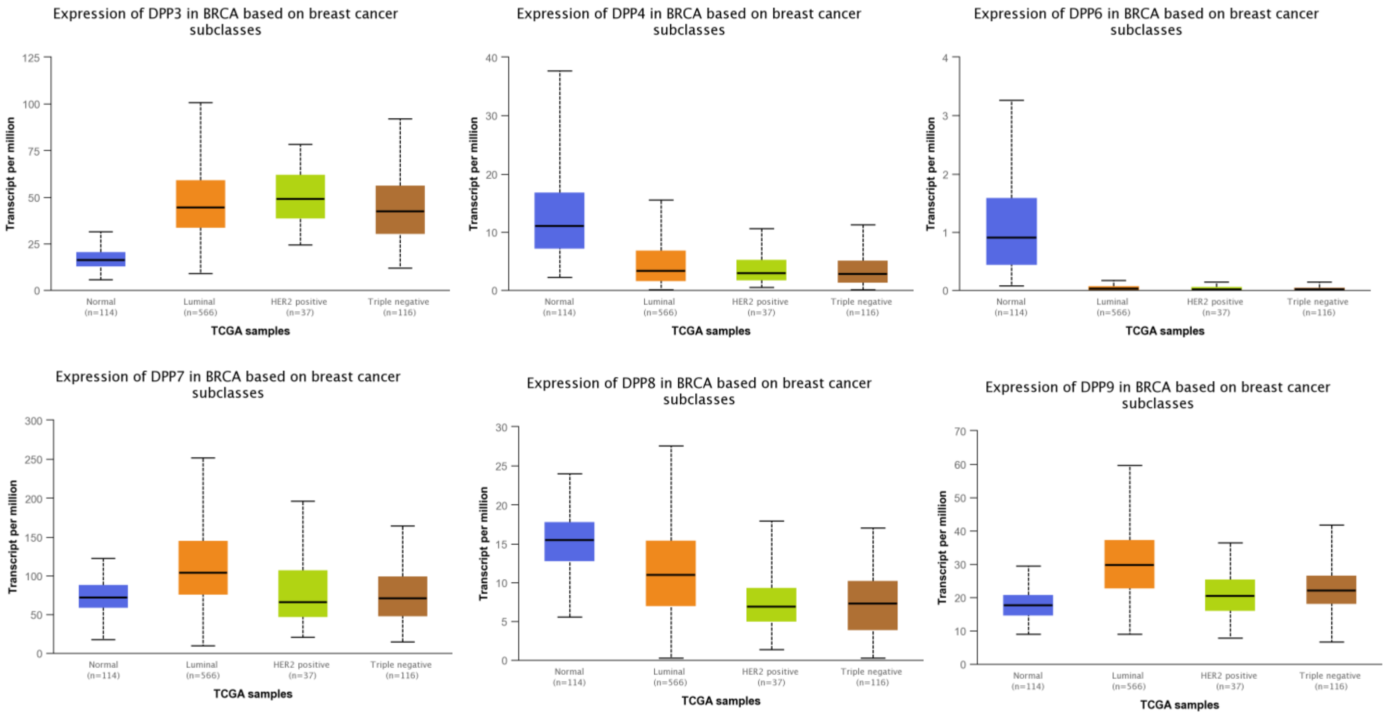

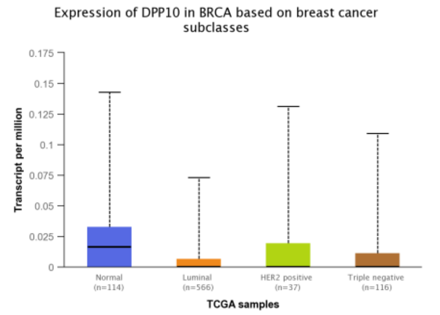

Comparison and P value	DPP3	DPP4	DPP6	DPP7	DPP8	DPP9	DPP10
Normal-vs-Luminal	<1E-12	1.63E-12	<1E-12	1.62E-12	5.72E-07	<1E-12	5.76E-04
Normal-vs-HER2 Positive	1.15E-08	3.16E-08	<1E-12	1.55E-01	6.40E-08	2.25E-02	8.24E-02
Normal-vs-TNBC	1.62E-12	6.78E-11	6.43E-04	3.70E-01	1.62E-12	7.03E-08	1.60E-03
Luminal-vs-HER2 Positive	1.75E-01	8.36E-01	2.64E-02	3.40E-01	7.48E-03	9.32E-12	5.05E-01
Luminal-vs-TNBC	3.10E-03	7.88E-01	1.39E-01	2.33E-15	1.38E-11	5.15E-10	7.39E-02
HER2 Positive-vs-TNBC	1.45E-02	7.36E-01	3.27E-02	2.39E-01	5.33E-01	3.60E-02	5.66E-01

Supplementary Figure S1. Transcription levels of dipeptidyl peptidase (*DPP*) family genes in different subtypes of breast cancer patients (TCGA database). Box plot showing relative expression levels of *DPP* family genes in normal, luminal, human epidermal growth factor receptor 2 (Her2), and triple-negative subtypes of breast cancer.

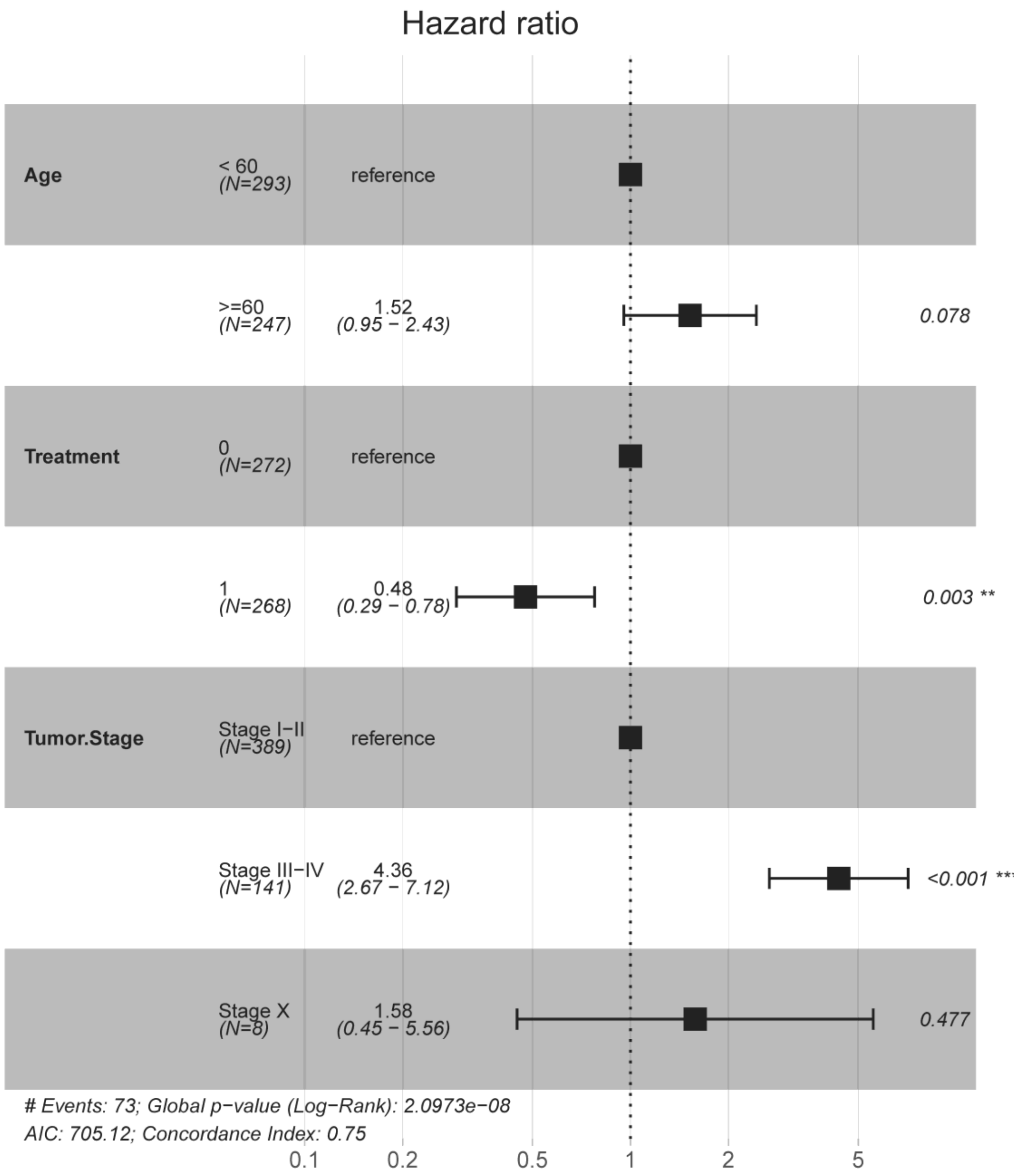

Supplementary Figure S2. Multivariate analysis of dipeptidyl peptidase 3 (DPP3) expression and relationships between it and clinicopathological parameters (age, treatment, stage, and TNM (tumor, node, metastasis) stage).

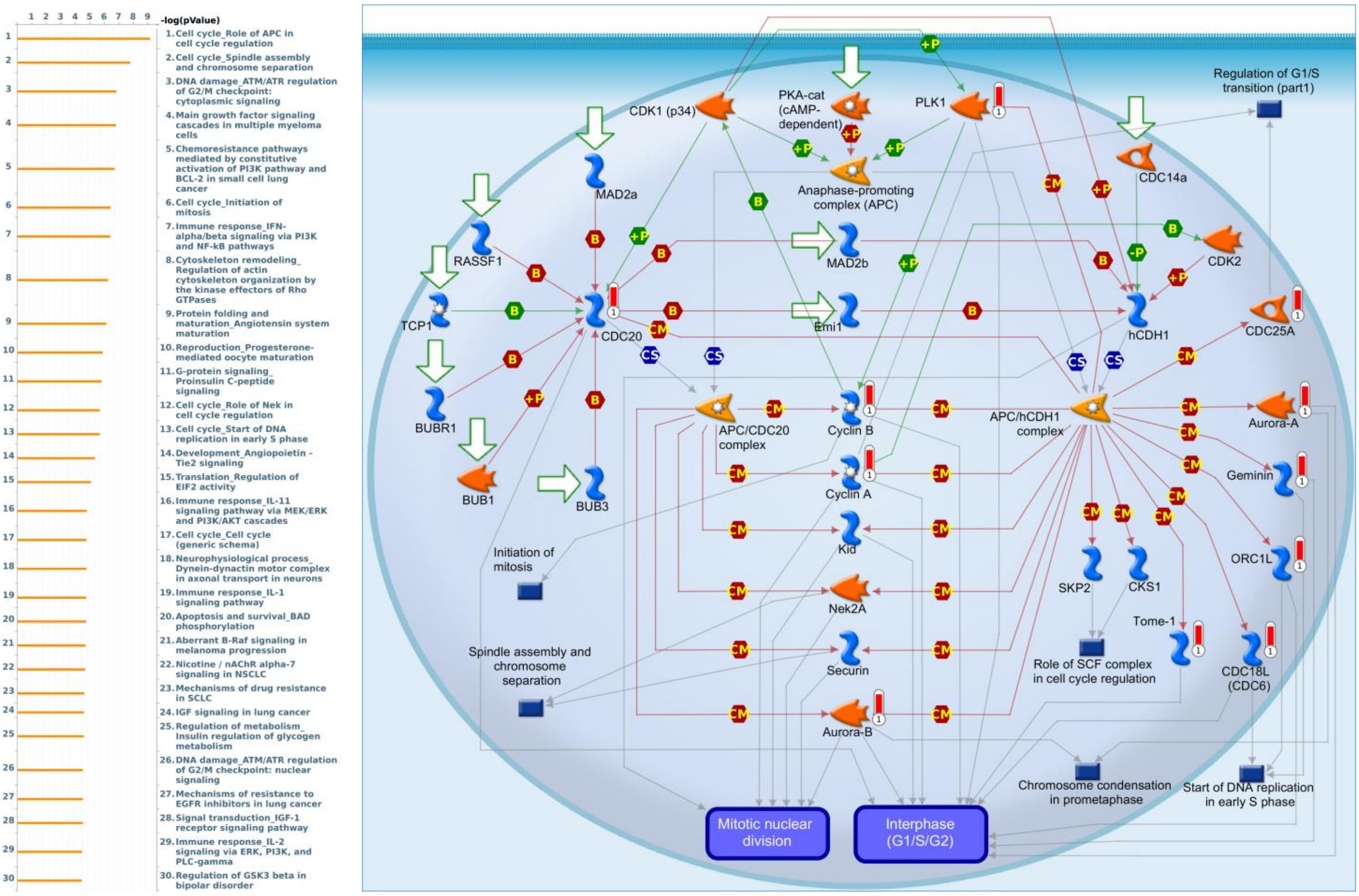

Supplementary Figure S3. MetaCore pathway analysis of the coexpression gene network of dipeptidyl peptidase 3 (DPP3) in breast cancer patients. Downstream pathway analyses revealed that "DPP3-Cell cycle_Role of APC in cell cycle regulation" participates in breast cancer development. Nodes represent individual proteins with different shapes for different functional classes of proteins. Interactions of nodes are illustrated by edges with arrows showing the direction of the integration. Green and red arrows respectively represent activation and inhibition. P represents phosphorylation, T represents transformation, B represents binding, C represents cleavage, and TR represents transcriptional regulation. A legend explaining the symbols used by MetaCore is provided at http://portal.genego.com/legends/legend_6.png.

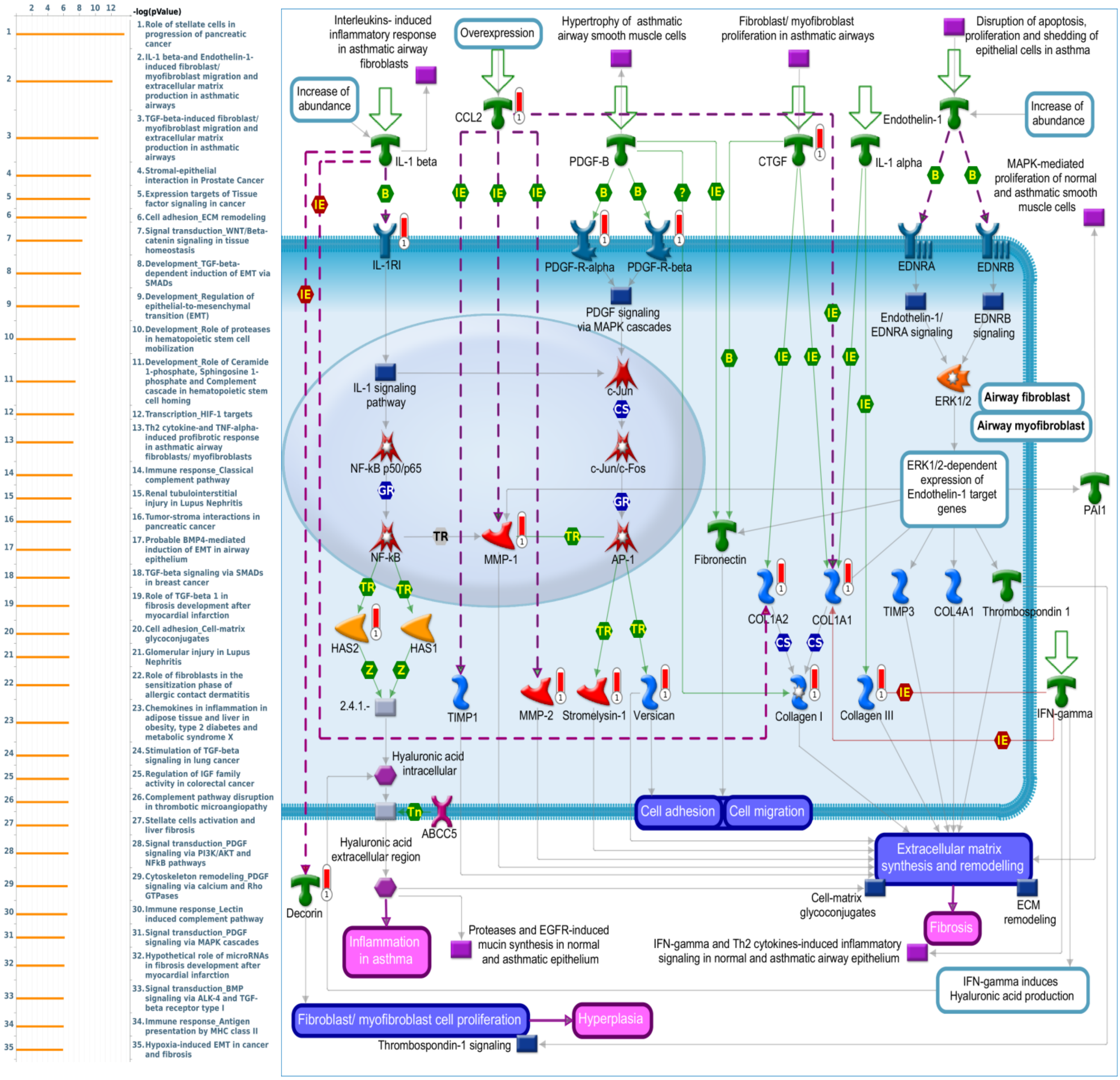

Supplementary Figure S4. MetaCore pathway analysis of the coexpression gene network of dipeptidyl peptidase 4 (DPP4) in breast cancer patients. Downstream pathway analyses revealed that "IL-1 beta- and Endothelin-1-induced fibroblast myofibroblast migration and extracellular matrix production in asthmatic airways" might participate in breast cancer development. Nodes represent individual proteins with different shapes for different functional classes of proteins. Interactions of nodes are illustrated by edges with arrows showing the direction of the integration. Green and red arrows respectively represent activation and inhibition. P represents phosphorylation, T represents transformation, B represents binding, C represents cleavage, and TR represents transcriptional regulation. A legend explaining the symbols used by MetaCore is provided at http://portal.genego.com/legends/legend_6.png.

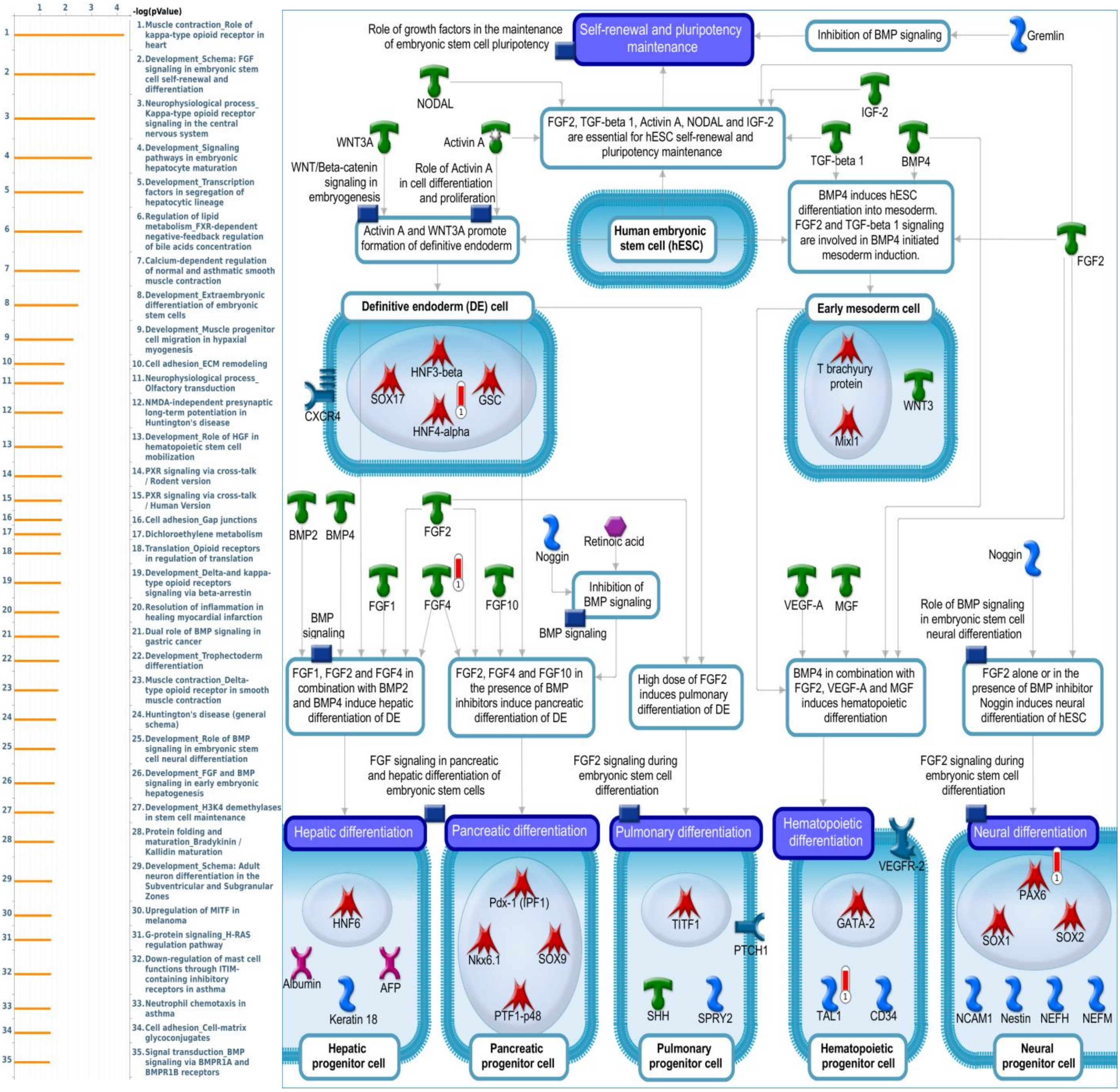

Supplementary Figure S5. MetaCore pathway analysis of the coexpression gene network of dipeptidyl peptidase 6 (DPP6) in breast cancer patients. Downstream pathway analyses revealed that "Development_Schema FGF signaling in embryonic stem cell self-renewal and differentiation" might be involved in breast cancer development. Nodes represent individual proteins with different shapes for different functional classes of proteins. Interactions of nodes are illustrated by edges with arrows showing the direction of the integration. Green and red arrows respectively represent activation and inhibition. P represents phosphorylation, T represents transformation, B represents binding, C represents cleavage, and TR represents transcriptional regulation. A legend explaining the symbols used by MetaCore is provided at http://portal.genego.com/legends/legend_6.png.

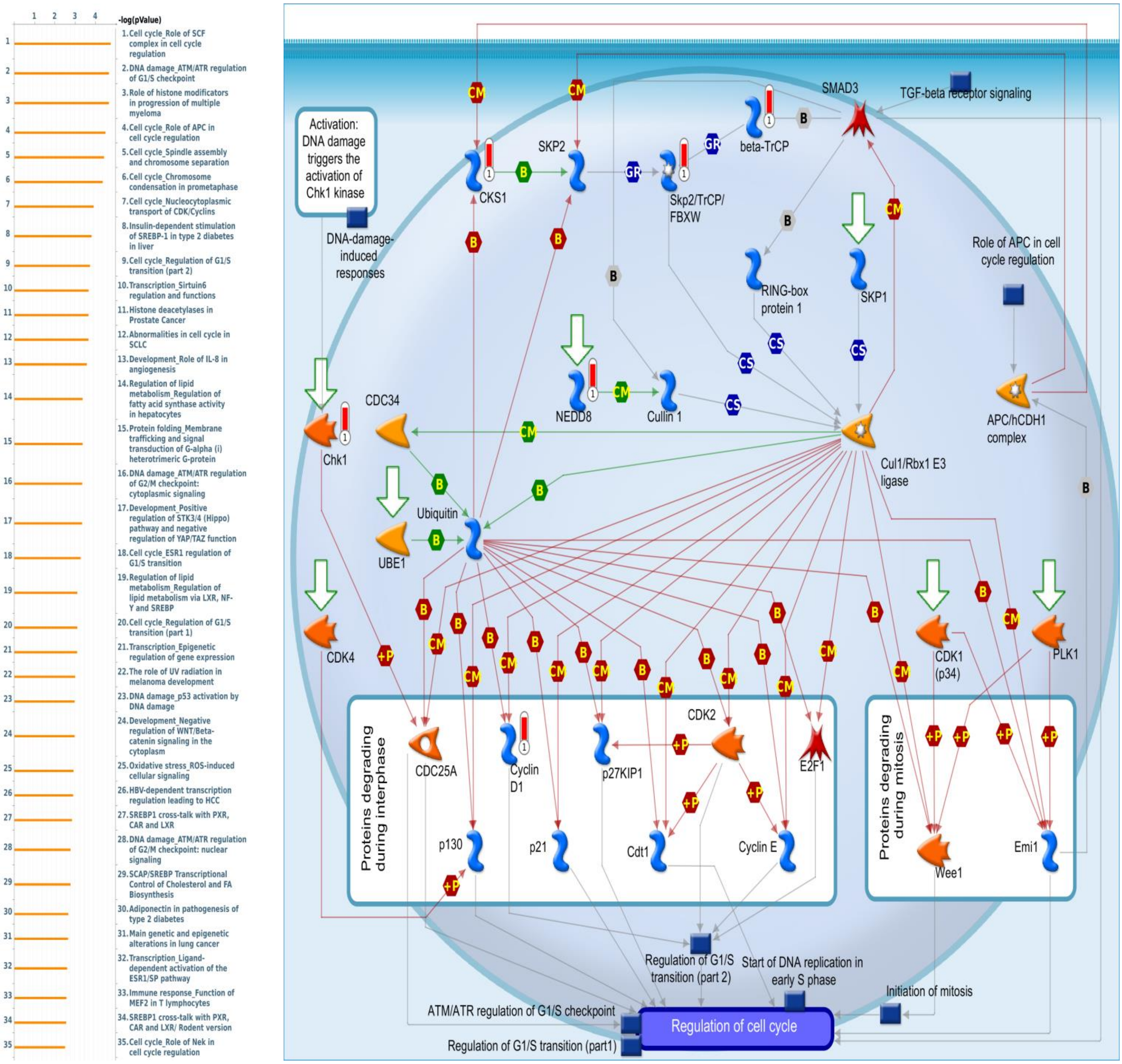

Supplementary Figure S6. MetaCore pathway analysis of the coexpression gene network of dipeptidyl peptidase 7 (DPP7) in breast cancer patients. Downstream pathway analyses revealed that "Cell cycle_Role of SCF complex in cell cycle regulation" might participate in breast cancer development. Nodes represent individual proteins with different shapes for different functional classes of proteins. Interactions of nodes are illustrated by edges with arrows showing the direction of the integration. Green and red arrows respectively represent activation and inhibition. P represents phosphorylation, T represents transformation, B represents binding C cleavage, and TR represents transcriptional regulation. A legend explaining the symbols used by MetaCore is provided at http://portal.genego.com/legends/legend_6.png.

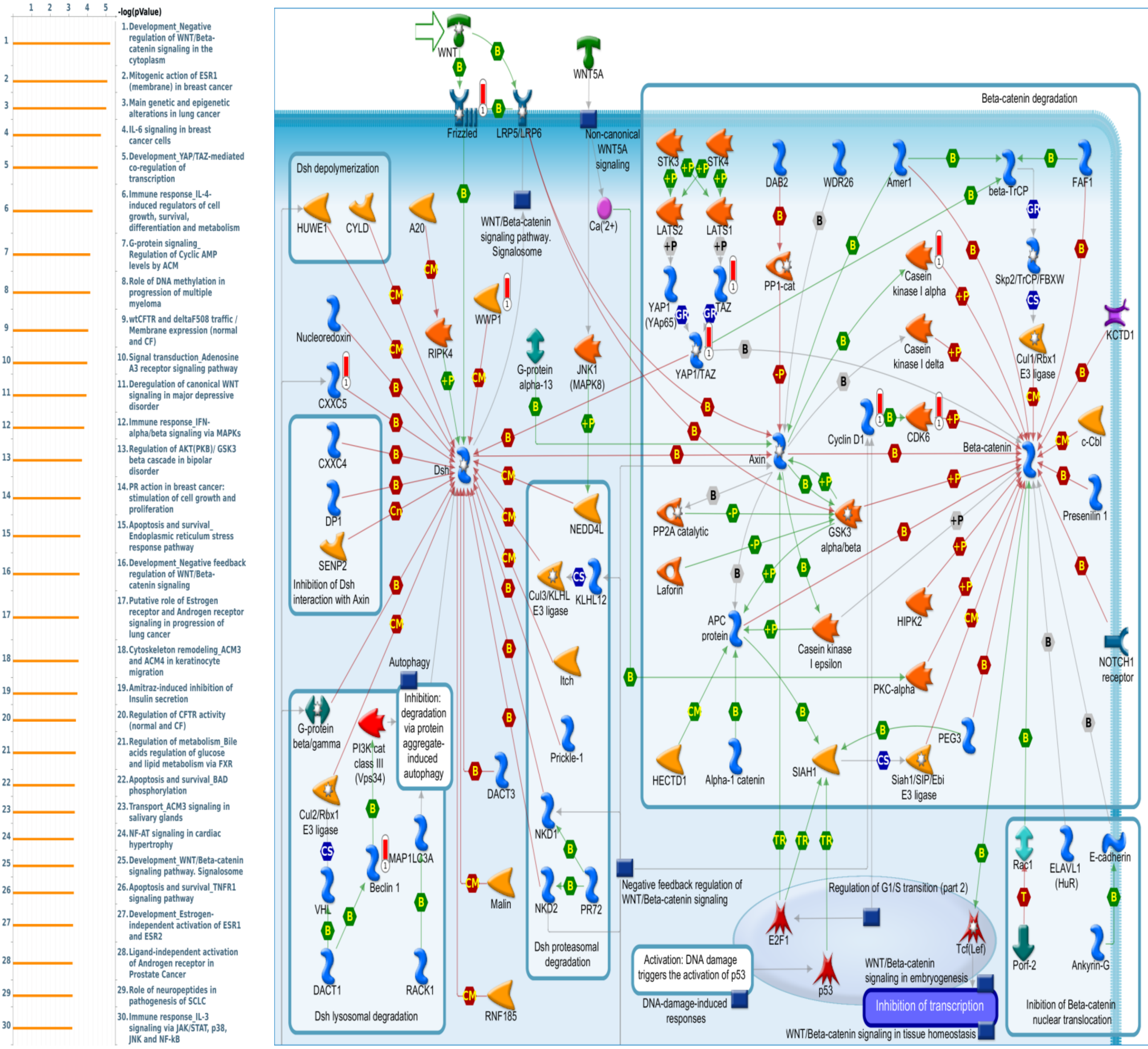

Supplementary Figure S7. MetaCore pathway analysis of the coexpression gene network of dipeptidyl peptidase 8 (DPP8) in breast cancer patients. Downstream pathway analyses revealed that "Development_Negative regulation of WNTBeta-catenin signaling in the cytoplasm" might be involved in breast cancer development. Nodes represent individual proteins with different shapes for different functional classes of proteins. Interaction of nodes are illustrated by edges with arrows showing the direction of the integration. Green and red arrows respectively represent activation and inhibition. P represents phosphorylation, T represents transformation, B represents binding, C represents cleavage, and TR represents transcriptional regulation. A legend explaining the symbols used by MetaCore is provided at http://portal.genego.com/legends/legend_6.png.

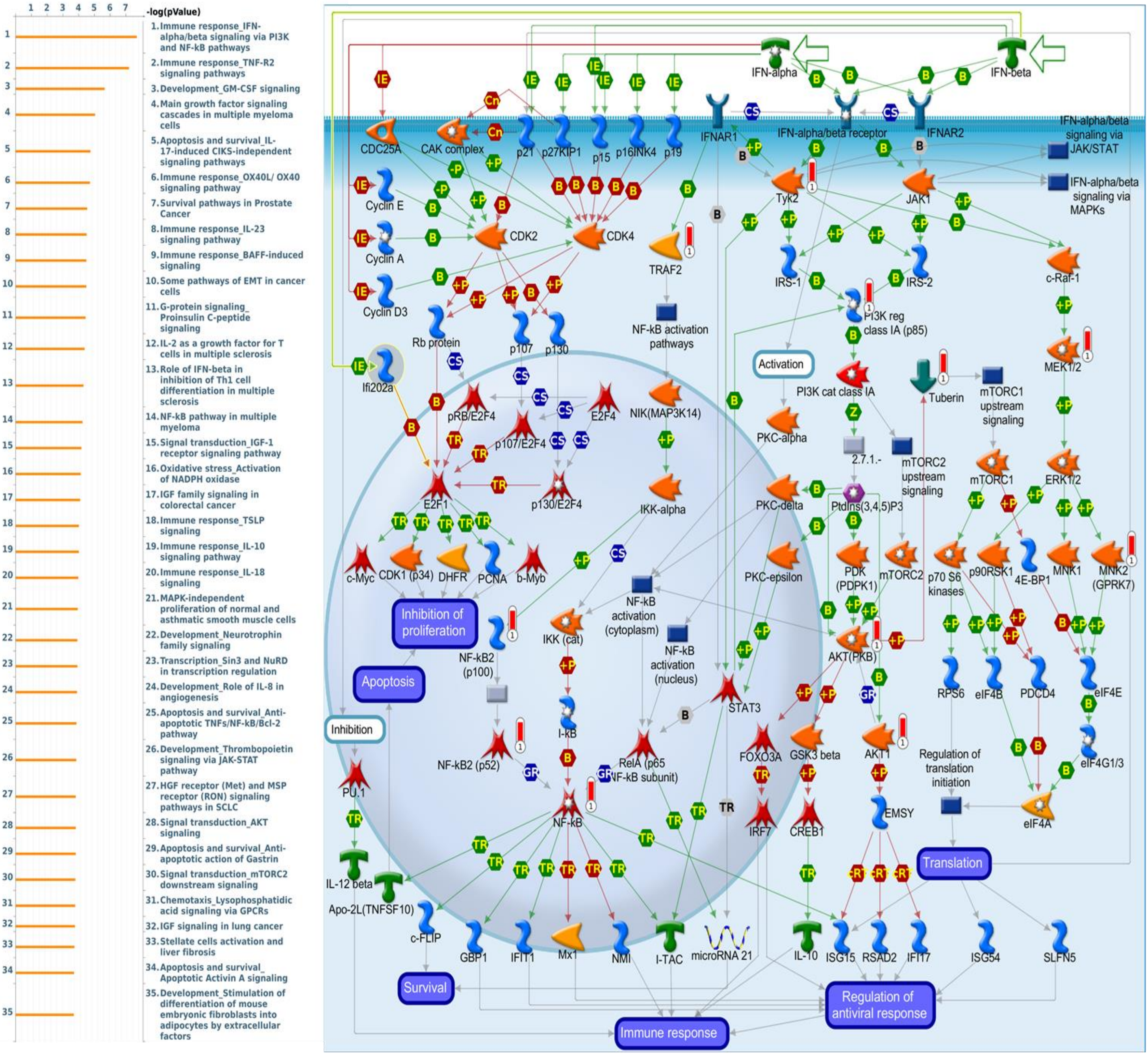

Supplementary Figure S8. MetaCore pathway analysis of the coexpression gene network of dipeptidyl peptidase 9 (DPP9) in breast cancer patients. Downstream pathway analyses revealed that "Immune response_IFN-alphabeta signaling via PI3K and NF-κB pathways" might participate in breast cancer development. Nodes represent individual proteins with different shapes for different functional classes of proteins. Interactions of nodes are illustrated by edges with arrows showing the direction of the integration. Green and red arrows respectively represent activation and inhibition. P represents phosphorylation, T represents transformation, B represents binding, C represents cleavage, and TR represents transcriptional regulation. A legend explaining the symbols used by MetaCore is provided at http://portal.genego.com/legends/legend_6.png.

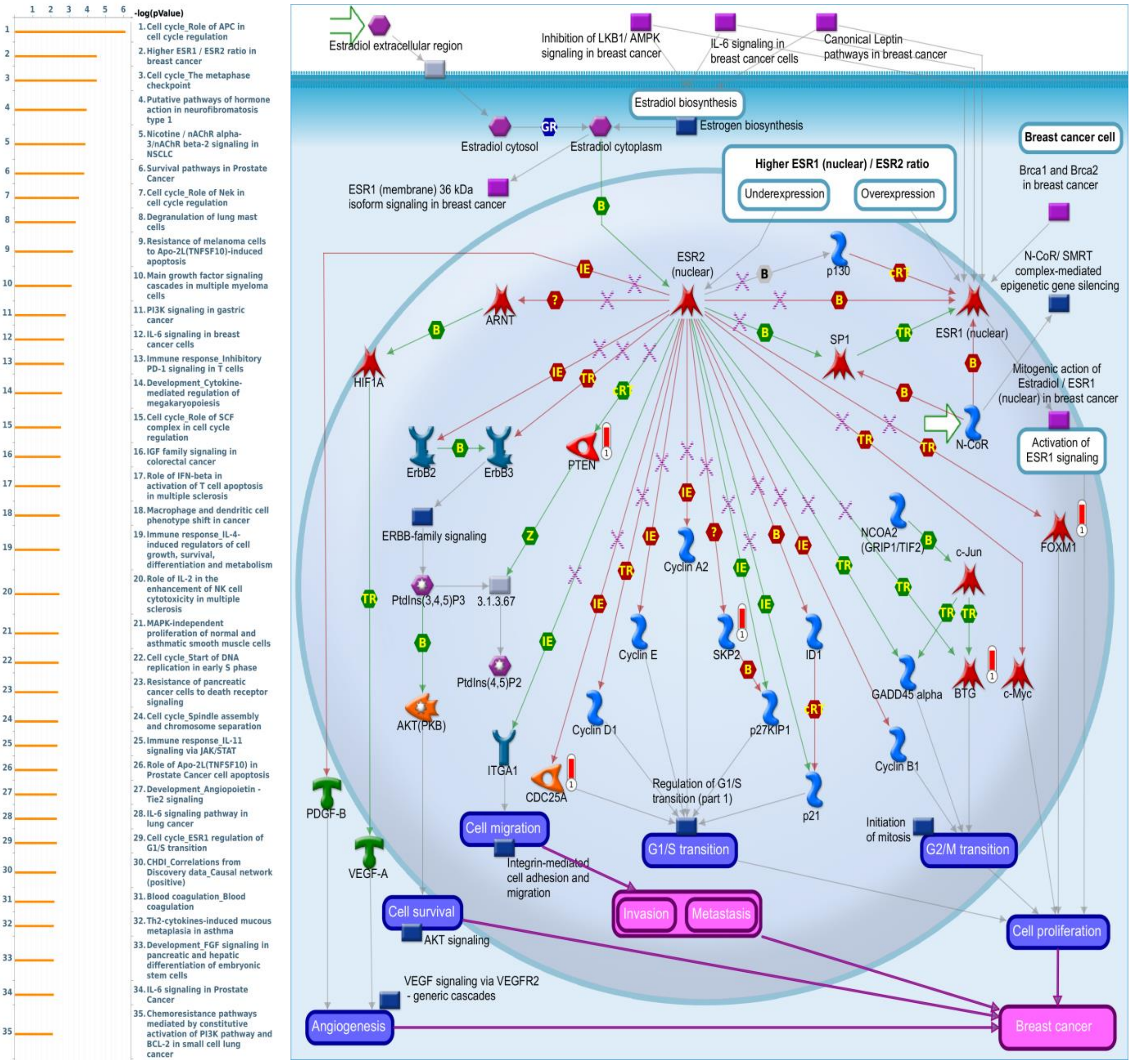

Supplementary Figure S9. MetaCore pathway analysis of the coexpression gene network of dipeptidyl peptidase 10 (DPP10) in breast cancer patients. Downstream pathway analyses revealed that "DPP10-Higher ESR1/ESR2 ratio in breast cancer" might participate in breast cancer development. Nodes represent individual proteins with different shapes for different functional classes of proteins. Interaction of nodes are illustrated by edges with arrows showing the direction of the integration. Green and red arrows respectively represent activation and inhibition. P represents phosphorylation, T represents transformation, B represents binding, C represents cleavage, and TR represents transcriptional regulation. A legend explaining the symbols used by MetaCore is provided at http://portal.genego.com/legends/legend_6.png.

Supplementary Table S1. Pathway analysis of dipeptidyl peptidase 3 (DPP3)-coexpressed genes from public breast cancer databases using the MetaCore database (with $p < 0.01$ set as the cut-off value).

#	Maps	*p*-Value	Network Objects from Active Data
**1**	Cell cycle_Role of APC in cell cycle regulation	6.93E-10	CDC18L (CDC6), Tome-1, Geminin, Cyclin A, Aurora-A, PLK1, Aurora-B, CDC25A, CDC20, Cyclin B, ORC1L
**2**	Cell cycle_Spindle assembly and chromosome separation	1.7E-08	Importin (karyopherin)-alpha, TPX2, CSE1L, Aurora-A, Aurora-B, CDC20, Tubulin alpha, Cyclin B, Separase, Tubulin (in microtubules)
**3**	DNA damage_ATM/ATR regulation of G2/M checkpoint: cytoplasmic signaling	1.57E-07	CDC25C, UBE2C, Cyclin B1, PP2A regulatory, Aurora-A, PLK1, PP1-cat, Aurora-B, MAPKAPK2, CDC25A, CDC25B
**4**	Main growth factor signaling cascades in multiple myeloma cells	1.68E-07	IRS-2, IGF-1, I-kB, PI3K reg class IA (p85), FGF2, H-Ras, MEK2(MAP2K2), PI3K reg class IA (p85-beta), AKT(PKB), PI3K reg class IA
**5**	Chemoresistance pathways mediated by constitutive activation of PI3K pathway and BCL-2 in small cell lung cancer	2.15E-07	MGF, IGF-1, p70 S6 kinase2, c-Kit, PI3K reg class IA (p85), RPS6, FGF2, Survivin, AKT(PKB), PI3K reg class IA
**6**	Cell cycle_Initiation of mitosis	4.19E-07	CDC25C, Cyclin B1, PLK1, Cyclin B2, CDC25B, FOXM1, Kinase MYT1, AKT(PKB)
**7**	Immune response_IFN-alpha/beta signaling via PI3K and NF-kB pathways	4.28E-07	IRS-2, I-kB, MEK1/2, TRAF2, Cyclin A, p70 S6 kinases, Cyclin E, PI3K reg class IA (p85), RPS6, AKT1, CDC25A, eIF4G1/3, AKT(PKB), ISG15
**8**	Cytoskeleton remodeling_Regulation of actin cytoskeleton organization by the kinase effectors of Rho GTPases	6.27E-07	ARPC1B, RhoJ, Cortactin, GIT1, MyHC, Rac3, Spectrin, MLCK, Rac1-related, RhoGDI alpha, Cdc42 subfamily
**9**	Protein folding and maturation_Angiotensin system maturation	8.18E-07	Angiotensin II, Angiotensin III, Angiotensin (2-10), Angiotensin (1-7), Angiotensinogen, Dnpep, Angiotensin (1-9), Angiotensin IV, Angiotensin I, Cathepsin A
**10**	Reproduction_Progesterone-mediated oocyte maturation	1.47E-06	CDC25C, Cyclin B1, Aurora-A, PLK1, H-Ras, CDC20, p90Rsk, CDC25B, Kinase MYT1
**11**	G-protein signaling_Proinsulin C-peptide signaling	1.79E-06	COX-2 (PTGS2), I-kB, TRAF2, PI3K reg class IA (p85), ATP1B1, H-Ras, IP3 receptor, MEK2(MAP2K2), AKT(PKB), PI3K reg class IA
**12**	Cell cycle_Role of Nek in cell cycle regulation	2.43E-06	Tubulin beta, Tubulin gamma, Cyclin B1, TPX2, Aurora-A, Tubulin alpha, PI3K reg class IA, Tubulin (in microtubules)
**13**	Cell cycle_Start of DNA replication in early S phase	2.43E-06	CDC18L (CDC6), Geminin, Cyclin E, MCM10, ORC6L, MCM2, ORC1L, CDC45L
**14**	Development_Angiopoietin - Tie2 signaling	5.04E-06	Angiopoietin 1, I-kB, Angiopoietin 3, Angiopoietin 2, Survivin, STAT1, AKT(PKB), PI3K reg class IA
**15**	Translation_Regulation of EIF2 activity	9.71E-06	IRS-2, MEK1/2, Casein kinase I, PP1-cat, H-Ras, PP1-cat alpha, AKT(PKB), PI3K reg class IA
**16**	Immune response_IL-11 signaling pathway via MEK/ERK and PI3K/AKT cascades	1.91E-05	NFKBIB, I-kB, MEK1/2, p70 S6 kinases, Cyclin E, PI3K reg class IA (p85), RPS6, H-Ras, p90Rsk, AKT(PKB)
**17**	Cell cycle_Cell cycle (generic schema)	2E-05	CDC25C, Cyclin A, Cyclin E, CDC25A, Cyclin B, CDC25B
**18**	Neurophysiological process_Dynein-dynactin motor complex in axonal transport in neurons	2.01E-05	Kinesin light chain, Importin (karyopherin)-alpha, Vimentin, Centractins, PRNP, SPTBN2, Alpha-centractin, AKT(PKB), Tubulin (in microtubules)

**19**	Immune response_IL-1 signaling pathway	2.08E-05	COX-2 (PTGS2), I-kB, MEK1/2, EGR1, JAM2, MYLK1, PI3K reg class IA (p85), FGF2, MAPKAPK2, STAT1, AKT(PKB)
**20**	Apoptosis and survival_BAD phosphorylation	2.12E-05	p70 S6 kinase2, H-Ras, p90Rsk, PP1-cat alpha, PP2C, MEK2(MAP2K2), AKT(PKB), PI3K reg class IA
**21**	Aberrant B-Raf signaling in melanoma progression	2.34E-05	SPRY2, MEK1/2, Aurora-B, AKT1, p90Rsk, FOXM1, Kinase MYT1, AKT(PKB), RhoE
**22**	Nicotine / nAChR alpha-7 signaling in NSCLC	2.54E-05	Calpain 1(mu), CACNA1C, Cyclin E, PI3K reg class IA (p85), H-Ras, p90Rsk, MEK2(MAP2K2), AKT(PKB)
**23**	Mechanisms of drug resistance in SCLC	2.83E-05	ABCC10, MGF, IGF-1, p70 S6 kinase2, c-Kit, RPS6, FGF2, Survivin, AKT(PKB), PI3K reg class IA
**24**	IGF signaling in lung cancer	3.03E-05	IRS-2, IGF-1, H-Ras, p90Rsk, Survivin, MEK2(MAP2K2), AKT(PKB), PI3K reg class IA
**25**	Regulation of metabolism_Insulin regulation of glycogen metabolism	3.15E-05	IRS-2, PHK gamma (liver), PP1-cat, H-Ras, GYS1, PHK gamma, MEK2(MAP2K2), AKT(PKB), PI3K reg class IA
**26**	DNA damage_ATM/ATR regulation of G2/M checkpoint: nuclear signaling	3.59E-05	CDC25C, CDC18L (CDC6), Cyclin B1, Cyclin A, CDC14b, PLK1, Cyclin B, Cyclin B2
**27**	Mechanisms of resistance to EGFR inhibitors in lung cancer	3.59E-05	HSP90, Vimentin, PI3K reg class IA (p85), H-Ras, Claudin-7, Survivin, MEK2(MAP2K2), AKT(PKB)
**28**	Signal transduction_IGF-1 receptor signaling pathway	3.64E-05	IRS-2, IGF-1, SCD, I-kB, MEK1/2, PI3K reg class IA (p85), RPS6, H-Ras, AKT(PKB)
**29**	Immune response_IL-2 signaling via ERK, PI3K, and PLC-gamma	4.11E-05	IRS-2, MEK1/2, p70 S6 kinase2, Cyclin A, p70 S6 kinases, Cyclin E, PI3K reg class IA (p85), RPS6, H-Ras, AKT(PKB)
**30**	Regulation of GSK3 beta in bipolar disorder	4.24E-05	MEK1/2, PP2A regulatory, Neutral sphingomyelinase, PP1-cat, FGF2, AKT1, H-Ras, AKT(PKB)
**31**	Neurogenesis_NGF/ TrkA MAPK-mediated signaling	4.54E-05	KIDINS220, MEK1/2, EGR1, RUSC1 (NESCA), PP2A regulatory, MAPKAPK2, H-Ras, p90Rsk, SORBS1, IP3 receptor, MEF2C, DNAJA3 (TID1)
**32**	IGF-1 receptor/EGFR cooperation in lung cancer	4.62E-05	IRS-2, IGF-1, p90Rsk, Survivin, AKT(PKB), PI3K reg class IA
**33**	IGF family signaling in colorectal cancer	4.8E-05	CDC25C, COX-2 (PTGS2), GIPC, IRS-2, IGF-1, I-kB, MEK1/2, H-Ras, AKT(PKB)
**34**	Pro-oncogenic action of Estradiol/ Estrogen receptors in ovarian cancer	4.8E-05	Leptin receptor, Cyclin A2, NCOA3 (pCIP/SRC3), I-kB, TRAP-1, Cyclin E, Survivin, AKT(PKB), PI3K reg class IA
**35**	Regulation of metabolism_Insulin signaling: generic cascades	4.98E-05	IRS-2, p70 S6 kinase2, RPS6, PP1-cat, H-Ras, MEK2(MAP2K2), AKT(PKB), PI3K reg class IA
**36**	Canonical Leptin pathways in breast cancer	4.98E-05	Leptin receptor, IRS-2, I-kB, MEK1/2, H-Ras, Survivin, AKT(PKB), PI3K reg class IA
**37**	Development_c-Kit ligand signaling pathway during hemopoiesis	5.49E-05	MGF, MEK1/2, c-Kit, PI3K reg class IA (p85), H-Ras, p90Rsk, STAT1, IP3 receptor, AKT(PKB)
**38**	Development_Glucocorticoid receptor signaling	5.93E-05	HSP90, GCR, FKBP4, E2I, GCR Alpha, GCR Beta
**39**	IGF-1 signaling in pancreatic cancer	6.11E-05	COX-2 (PTGS2), IRS-2, IGF-1, AKT1, H-Ras, MEK2(MAP2K2), PI3K reg class IA
**40**	Cell cycle_The metaphase checkpoint	6.11E-05	SPBC25, Aurora-A, PLK1, Aurora-B, CDC20, HZwint-1, Survivin

**41**	Development_VEGF signaling via VEGFR2 - generic cascades	6.85E-05	HSP90, COX-2 (PTGS2), I-kB, MAPKAPK2, H-Ras, p90Rsk, MLCK, IP3 receptor, MEK2(MAP2K2), AKT(PKB), PI3K reg class IA
**42**	IGF-1 signaling in multiple myeloma	7.9E-05	IRS-2, IGF-1, I-kB, MEK1/2, H-Ras, Survivin, AKT(PKB), PI3K reg class IA
**43**	Anti-apoptotic action of ErbB2 in breast cancer	9.14E-05	Calpain 1(mu), I-kB, MEK1/2, PI3K reg class IA (p85), AKT1, H-Ras, Survivin, AKT(PKB)
**44**	Role of tumor microenvironment in plexiform neurofibroma formation in neurofibromatosis type 1	0.000104	MGF, MEK1/2, c-Kit, PI3K reg class IA (p85), FGF2, H-Ras, AKT(PKB)
**45**	Regulation of degradation of deltaF508-CFTR in CF	0.000104	HSP90, Csp, Sti1, Aha1, SAE1, E2I, NPL4
**46**	Development_Role of growth factors in the maintenance of embryonic stem cell pluripotency	0.000121	IGF-1, PI3K reg class IA (p85), FGF2, H-Ras, SMAD4, MEK2(MAP2K2), AKT(PKB), PI3K reg class IA
**47**	Cell cycle_Influence of Ras and Rho proteins on G1/S Transition	0.000121	Cyclin A2, MLK3(MAP3K11), Cyclin E, H-Ras, MLCK, MEK2(MAP2K2), AKT(PKB), PI3K reg class IA
**48**	Transcription_Role of heterochromatin protein 1 (HP1) family in transcriptional silencing	0.000124	Cyclin A2, TIF1-beta, Mi-2, Cyclin E, E2I, CDC25A, MEF2
**49**	Immune response_Gastrin in inflammatory response	0.000131	COX-2 (PTGS2), I-kB, PI3K reg class IA (p85), H-Ras, MEF2, IP3 receptor, MEK2(MAP2K2), MEF2C, AKT(PKB)
**50**	Development_FGFR signaling pathway	0.000139	MLK3(MAP3K11), FGF2, MAPKAPK2, H-Ras, IP3 receptor, MEK2(MAP2K2), AKT(PKB), PI3K reg class IA

Supplementary Table S2. Pathway analysis of dipeptidyl peptidase 4 (DPP4)-coexpressed genes from public breast cancer databases using the MetaCore database (with $p < 0.01$ set as the cut-off value).

#	**Maps**	***p*-Value**	**Network Objects from Active Data**
**1**	Role of stellate cells in progression of pancreatic cancer	2.19E-14	COL1A1, PDGF receptor, OSF-2, GRO-2, IL-6, Galectin-1, CCL2, ACTA2, Stromelysin-1, TGF-beta receptor type II, RECK, MMP-13, COL1A2, Collagen I, CTGF, PDGF-R-alpha, PDGF-R-beta, MMP-2, Collagen III
**2**	IL-1 beta- and Endothelin-1-induced fibroblast/ myofibroblast migration and extracellular matrix production in asthmatic airways	7.28E-13	COL1A1, IL-1RI, CCL2, MMP-1, Stromelysin-1, HAS2, COL1A2, Collagen I, CTGF, PDGF-R-alpha, PDGF-R-beta, MMP-2, Versican, Collagen III, Decorin
**3**	TGF-beta-induced fibroblast/ myofibroblast migration and extracellular matrix production in asthmatic airways	4.81E-11	COL1A1, TIMP2, TGF-beta 3, ITGB1, MMP-1, Stromelysin-1, HAS2, TGF-beta receptor type II, MMP-13, COL1A2, Collagen I, COL5A1, MMP-2, Collagen III, Thrombospondin 2, Decorin
**4**	Stromal-epithelial interaction in Prostate Cancer	4.14E-10	HIC5, TIMP2, IL-6, IGF-1, TGF-beta 3, ACTA2, Vimentin, TGF-beta receptor type II, Collagen I, PDGF-R-alpha, PDGF-R-beta, MMP-2, Versican
**5**	Expression targets of Tissue factor signaling in cancer	5.48E-10	Tissue factor, GFPT2, ITGB1, PLAUR (uPAR), PAR2, Coagulation factor X, PLAU (UPA), VEGF-C, CTGF, Cyr61

**6**	Cell adhesion_ECM remodeling	1.56E-09	MMP-12, TIMP2, Caveolin-2, IGF-1, PLAUR (uPAR), MMP-1, Stromelysin-1, LAMA4, MMP-13, PLAU (UPA), Collagen I, MMP-2, Versican, Collagen III
**7**	Signal transduction_WNT/Beta-catenin signaling in tissue homeostasis	5.53E-09	COX-2 (PTGS2), Cyclin D2, Cyclin A2, FKHR, BACE1, Tcf(Lef), WNT, SLUG, Pitx2, TCF7L2 (TCF4), MMP-2, Versican
**8**	Development_TGF-beta-dependent induction of EMT via SMADs	7.88E-09	TGF-beta 3, Vimentin, TCF8, TGF-beta, ETS1, TGF-beta receptor type II, SLUG, ILK, TWIST1, SIP1 (ZFHX1B), MMP-2
**9**	Development_Regulation of epithelial-to-mesenchymal transition (EMT)	1.31E-08	IL-1RI, TGF-beta 3, ACTA2, Caldesmon, Vimentin, TCF8, WNT, TGF-beta receptor type II, SLUG, PDGF-R-alpha, TWIST1, SIP1 (ZFHX1B), PDGF-R-beta, MMP-2
**10**	Development_Role of proteases in hematopoietic stem cell mobilization	3.88E-08	MGF, c-Kit, Cathepsin G, VCAM1, Cathepsin K, SDF-1, CXCR4, MMP-2
**11**	Development_Role of Ceramide 1-phosphate, Sphingosine 1-phosphate and Complement cascade in hematopoietic stem cell homing	4.08E-08	COX-2 (PTGS2), PGE2R2, PGE2R4, C3a, VCAM1, SDF-1, C3aR, iC3b, CXCR4, C1qRp
**12**	Transcription_HIF-1 targets	6.24E-08	ROR-alpha, Galectin-1, Adrenomedullin, MGF, TGF-beta 3, PLAUR (uPAR), Adipophilin, 5'-NTD, GLUT3, SDF-1, CXCR4, LRP1, CTGF, Lysyl oxidase, IBP3, MMP-2
**13**	Th2 cytokine- and TNF-alpha-induced profibrotic response in asthmatic airway fibroblasts/ myofibroblasts	7.68E-08	COL1A1, COX-2 (PTGS2), ACTA2, MMP-1, HAS2, ETS1, COL1A2, Collagen I, PDGF-R-alpha, MMP-2, Collagen III, Decorin
**14**	Immune response_Classical complement pathway	9.64E-08	DAF, C1 inhibitor, C3a, C3, Factor I, C3b, C1s, C3aR, iC3b, C1qRp, C3dg, C3c
**15**	Renal tubulointerstitial injury in Lupus Nephritis	1.35E-07	IL-6, CCL2, Vimentin, HAS2, MHC class II, VCAM1, PLAU (UPA), FN14(TNFRSF12A), Collagen I, BAFF(TNFSF13B), TIG2, SOCS1, Collagen III
**16**	Tumor-stroma interactions in pancreatic cancer	1.5E-07	OSF-2, Galectin-3, IGF-1, MMP-1, COL1A2, Collagen I, PDGF-R-beta, MMP-2, Thrombospondin 2
**17**	Probable BMP4-mediated induction of EMT in airway epithelium	1.66E-07	COL1A1, TCF8, SLUG, TWIST1, SIP1 (ZFHX1B), COL5A1, MMP-2, ALK-2
**18**	TGF-beta signaling via SMADs in breast cancer	2.31E-07	NOX4, TGF-beta 3, ATF-3, ETS1, TGF-beta receptor type II, SLUG, MMP-13, MTS1 (S100A4), CTGF, TWIST1, RUNX2
**19**	Role of TGF-beta 1 in fibrosis development after myocardial infarction	2.51E-07	COL1A1, TIMP2, ACTA2, MMP-1, TGF-beta receptor type II, COL1A2, Collagen I, CTGF, MMP-2, Collagen III
**20**	Cell adhesion_Cell-matrix glycoconjugates	2.51E-07	LYVE-1, Galectin-3, TIMP2, MMP-1, Stromelysin-1, Elastin, Fibulin-2, Fibulin-1, Neurocan, Versican
**21**	Glomerular injury in Lupus Nephritis	2.52E-07	OX40L(TNFSF4), GRO-2, IL-6, CCL2, PKC-alpha, C3a, MMP-1, TGF-beta, HAS2, GRO-3, VCAM1, FN14(TNFRSF12A), FasR(CD95), PDGF-R-beta, Decorin

**22**	Role of fibroblasts in the sensitization phase of allergic contact dermatitis	2.53E-07	IL-1RI, IL-6, Elastin, SDF-1, CXCR4, Collagen I, MMP-2, Collagen III
**23**	Chemokines in inflammation in adipose tissue and liver in obesity, type 2 diabetes and metabolic syndrome X	2.91E-07	LYVE-1, IL-6, CCL2, PLAUR (uPAR), MHC class II, P-selectin, VCAM1, CXCR4, CD14, CD1c, TIG2
**24**	Stimulation of TGF-beta signaling in lung cancer	2.91E-07	COX-2 (PTGS2), TGF-beta 3, ITGB1, ACTA2, Vimentin, TGF-beta, Fyn, TGF-beta receptor type II, SLUG, Tropomyosin-2, MMP-2
**25**	Regulation of IGF family activity in colorectal cancer	2.94E-07	COX-2 (PTGS2), IBP6, IL-6, IGF-1, PLAUR (uPAR), PCSK5, IBP, PLAU (UPA), IBP3
**26**	Complement pathway disruption in thrombotic microangiopathy	3.28E-07	Factor H, MYLK1, C3a, C3, cPKC (conventional), Factor I, P-selectin, C3b, C3aR, PKC
**27**	Stellate cells activation and liver fibrosis	3.36E-07	COL1A1, PDGF receptor, IL-1RI, GRO-2, CCL2, ACTA2, Tcf(Lef), TGF-beta receptor type II, COL1A2, KLF6, PDGF-R-alpha, PDGF-R-beta, MMP-2
**28**	Signal transduction_PDGF signaling via PI3K/AKT and NFkB pathways	3.36E-07	PDGF receptor, PDGF-C, FKHR, CCL2, Transgelin, ACTA2, SGK1, ETS1, PDGFR-ab, Phox1 (PRRX1), PDGF-R-alpha, PDGF-R-beta, MMP-2
**29**	Cytoskeleton remodeling_PDGF signaling via calcium and Rho GTPases	4.12E-07	PDGF receptor, CACNA1C, PDGF-C, PKC-alpha, Fyn, PDGFR-ab, ARPC2, Lysyl oxidase, PKC, PDGF-R-alpha, WaspIP, NCK1, PDGF-R-beta, DOCK8
**30**	Immune response_Lectin induced complement pathway	4.54E-07	DAF, C1 inhibitor, C3a, C3, Factor I, C3b, C3aR, iC3b, C1qRp, C3dg, C3c
**31**	Signal transduction_PDGF signaling via MAPK cascades	1.02E-06	PDGF receptor, COX-2 (PTGS2), Tissue factor, PDGF-C, IL-6, Stromelysin-1, PDGFR-ab, MMP-13, Phox1 (PRRX1), PDGF-R-alpha, PDGF-R-beta, MMP-2
**32**	Hypothetical role of microRNAs in fibrosis development after myocardial infarction	1.1E-06	COL1A1, Fibrillin 1, TGF-beta receptor type II, COL1A2, Collagen I, CTGF, MMP-2, Collagen III
**33**	Signal transduction_BMP signaling via ALK-4 and TGF-beta receptor type I	1.32E-06	COX-2 (PTGS2), ActRIIA, MGF, HAS2, CTGF, Cyr61, ActRIIB
**34**	Immune response_Antigen presentation by MHC class II	1.33E-06	MHC class II alpha chain, LLIR, Cathepsin L, MHC class II beta chain, Dectin-1, PKC-alpha, Fc gamma RII beta, Fc epsilon RI gamma, Cathepsin V, MYO1E, MHC class II, CLEC10A, OLR1, Legumain, LRP1, PKC
**35**	Hypoxia-induced EMT in cancer and fibrosis	1.62E-06	TCF8, ETS1, SLUG, Lysyl oxidase, TWIST1, SIP1 (ZFHX1B)
**36**	Bone metastases in Prostate Cancer	2.78E-06	PTHR1, CCL2, IGF-1, WNT, SDF-1, CXCR4, RUNX2, IBP3
**37**	Rheumatoid arthritis (general schema)	3.84E-06	MHC class II beta chain, IL-6, MMP-1, Stromelysin-1, TGF-beta, LFA-3, MHC class II, VCAM1, PTPN22, BAFF(TNFSF13B)
**38**	IL-1 signaling in melanoma	6.47E-06	COX-2 (PTGS2), IL-1RI, GRO-2, IL-6, CCL2, MMP-1, GRO-3, MITF, VCAM1
**39**	Proinflammatory cytokine production by Th17 cells in asthma	6.69E-06	ROR-alpha, COX-2 (PTGS2), IL-1RI, IL-6, IRF4, C3a, C3, MHC class II, C3aR, CXCR4

**40**	Immune response_Alternative complement pathway	6.69E-06	DAF, Factor H, C3a, C3, Factor I, C3b, C3aR, iC3b, C3dg, C3c
**41**	TGF-beta 1-mediated induction of EMT in normal and asthmatic airway epithelium	9.71E-06	COL1A1, ACTA2, Vimentin, ETS1, TGF-beta receptor type II, SLUG, CTGF, TWIST1, MMP-2
**42**	Eosinophil adhesion and transendothelial migration in asthma	1.1E-05	Galectin-3, MGF, c-Kit, PLAUR (uPAR), C3a, P-selectin, VCAM1, C3aR, PLAU (UPA), PKC, CCL13
**43**	Interleukins-induced inflammatory response in asthmatic airway fibroblasts	1.28E-05	COX-2 (PTGS2), IL-1RI, GRO-2, IL-6, MGF, CCL2, VCAM1, IL-33
**44**	Role of adhesion of SCLC cells in tumor progression	1.28E-05	ITGB1, Stromelysin-1, P-selectin, VCAM1, SDF-1, CXCR4, MMP-2, Caveolin-1
**45**	Extracellular matrix-regulated proliferation of airway smooth muscle cells in asthma	1.28E-05	COL1A1, TGF-beta receptor type II, Fibulin-1, LAMA1, Collagen I, CTGF, Collagen III, Decorin
**46**	Development_TGF-beta-dependent induction of EMT via RhoA, PI3K and ILK	1.43E-05	HIC5, TGF-beta 3, ACTA2, Caldesmon, Vimentin, Actin, TGF-beta receptor type II, SLUG, ILK
**47**	Down-regulation of mast cell functions through ITIM-containing inhibitory receptors in asthma	1.99E-05	MHC class I, MGF, CCL2, SHPS-1, c-Kit, Fc epsilon RI gamma, Fyn, Fc epsilon RI beta
**48**	Mast cell migration in asthma	2.21E-05	PDGF receptor, MGF, CCL2, TGF-beta 3, c-Kit, C3a, TGF-beta receptor type II, SDF-1, C3aR, CXCR4, Collagen I
**49**	TGF-beta 1-induced transactivation of membrane receptors signaling in HCC	2.89E-05	PDGF receptor, ITGB1, Cyclin A, TGF-beta, Actin, TGF-beta receptor type II, SLUG, PDGF-R-alpha, PDGF-R-beta
**50**	Vascular endothelial cell damage in SLE	3.28E-05	IL-1RI, Tissue factor, IL-6, CCL2, Caspase-1, C3a, MSR1, P-selectin, VCAM1, FN14(TNFRSF12A)

Supplementary Table S3. Pathway analysis of dipeptidyl peptidase 6 (DPP6)-coexpressed genes from public breast cancer databases using the MetaCore database (with $p < 0.01$ set as the cut-off value).

#	Maps	*p*-Value	Network Objects from Active Data
**1**	Muscle contraction_Role of kappa-type opioid receptor in heart	5.38E-05	Dynorphin B, Dynorphin A(1-13), DYN-A(1-8), Proenkephalin-B, DYN-A(1-17)
**2**	Development_Schema: FGF signaling in embryonic stem cell self-renewal and differentiation	7.43E-04	HNF4-alpha, TAL1, PAX6, FGF4
**3**	Neurophysiological process_Kappa-type opioid receptor signaling in the central nervous system	7.51E-04	Dynorphin B, Dynorphin A(1-13), DYN-A(1-8), Proenkephalin-B, DYN-A(1-17)
**4**	Development_Signaling pathways in embryonic hepatocyte maturation	1.01E-03	PXR, HNF1-alpha, HGF receptor (Met), HNF4-alpha

**5**	Development_Transcription factors in segregation of hepatocytic lineage	2.23E-03	HNF1-alpha, HGF receptor (Met), HNF4-alpha
**6**	Regulation of lipid metabolism_FXR-dependent negative-feedback regulation of bile acids concentration	2.46E-03	PXR, FGF19, HNF4-alpha
**7**	Calcium-dependent regulation of normal and asthmatic smooth muscle contraction	3.11E-03	BDNF, MRLC, Neurokinin B, Ryanodine receptor 3
**8**	Development_Extraembryonic differentiation of embryonic stem cells	3.49E-03	SMAD9 (SMAD8), PAX6, HNF4
**9**	Development_Muscle progenitor cell migration in hypaxial myogenesis	5.47E-03	HGF receptor (Met), PAX3, FGF4
**10**	Cell adhesion_ECM remodeling	1.23E-02	Kallikrein 1, MMP-16, Kallikrein 3 (PSA)
**11**	Neurophysiological process_Olfactory transduction	1.30E-02	Olfactory receptor, G-protein alpha-olf
**12**	NMDA-independent presynaptic long-term potentiation in Huntington's disease	1.42E-02	BDNF, GluR5, Complexin 2
**13**	Development_Role of HGF in hematopoietic stem cell mobilization	1.43E-02	HGF receptor (Met), G-CSF
**14**	PXR signaling via cross-talk / Rodent version	1.57E-02	PXR, HNF4-alpha
**15**	PXR signaling via cross-talk / Human Version	1.57E-02	PXR, HNF4-alpha
**16**	Cell adhesion_Gap junctions	1.57E-02	Tubulin beta, Connexin 45
**17**	Dichloroethylene metabolism	1.71E-02	HNF1-alpha, HNF4-alpha
**18**	Translation_Opioid receptors in regulation of translation	1.71E-02	Leu-enkephalin, Dynorphin A(1-13)
**19**	Development_Delta- and kappa-type opioid receptors signaling via beta-arrestin	1.71E-02	Leu-enkephalin, Dynorphin A(1-13)
**20**	Resolution of inflammation in healing myocardial infarction	2.00E-02	HGF receptor (Met), G-CSF
**21**	Dual role of BMP signaling in gastric cancer	2.00E-02	LI-cadherin, SMAD9 (SMAD8)
**22**	Development_Trophectoderm differentiation	2.00E-02	FGF4, ITGA7
**23**	Muscle contraction_Delta-type opioid receptor in smooth muscle contraction	2.15E-02	Leu-enkephalin, MRLC
**24**	Huntington's disease (general schema)	2.60E-02	BDNF
**25**	Development_Role of BMP signaling in embryonic stem cell neural differentiation	2.82E-02	SMAD9 (SMAD8), IRX1

**26**	Development_FGF and BMP signaling in early embryonic hepatogenesis	3.00E-02	HNF1-alpha, HNF4-alpha
**27**	Development_H3K4 demethylases in stem cell maintenance	3.18E-02	Na(v) I alpha, FGF4
**28**	Protein folding and maturation_Bradykinin / Kallidin maturation	3.18E-02	Tissue kallikreins, CPB2
**29**	Development_Schema: Adult neuron differentiation in the Subventricular and Subgranular Zones	3.75E-02	BDNF, GDNF
**30**	Upregulation of MITF in melanoma	3.95E-02	HGF receptor (Met), IMP1(ZBP1)
**31**	G-protein signaling_H-RAS regulation pathway	4.15E-02	GDNF, DOK2
**32**	Down-regulation of mast cell functions through ITIM-containing inhibitory receptors in asthma	4.15E-02	DOK2, OX-2 receptor 1
**33**	Neutrophil chemotaxis in asthma	4.36E-02	Tissue kallikreins, PTAFR
**34**	Cell adhesion_Cell-matrix glycoconjugates	4.36E-02	BCAN, ITIH
**35**	Signal transduction_BMP signaling via BMPR1A and BMPR1B receptors	4.56E-02	BMP5, SMAD9 (SMAD8)
**36**	Blood coagulation_Blood coagulation	4.56E-02	CPB2, Coagulation factor XI
**37**	Development_FGF signaling in pancreatic and hepatic differentiation of embryonic stem cells	4.78E-02	HNF4-alpha, FGF4
**38**	Development_Regulation of lung epithelial progenitor cell differentiation	5.00E-02	SMAD9 (SMAD8), SP-C
**39**	Regulation of metabolism_Bile acids regulation of glucose and lipid metabolism via FXR	5.00E-02	HNF1-alpha, HNF4-alpha
**40**	Noise-induced hair cell death and spiral ganglion neuron degeneration	5.20E-02	CACNA1G, BDNF, GDNF
**41**	Transcription_FXR-regulated cholesterol and bile acids cellular transport	5.22E-02	PXR, HNF4-alpha
**42**	Signal transduction_WNT/Beta-catenin signaling in tissue homeostasis	5.22E-02	CACNA1G, BDNF
**43**	Inhibition of remyelination in multiple sclerosis: regulation of cytoskeleton proteins	5.67E-02	Tubulin beta, MRLC
**44**	TMPRSS2-ERG fusion in Prostate Cancer	6.13E-02	TAL1, Kallikrein 3 (PSA)
**45**	Neurophysiological process_ACM regulation of nerve impulse	6.61E-02	CACNA1G, CACNA1H
**46**	L-Threonine metabolism	7.10E-02	ASCT2 (SLC1A5), SLC38A4

**47**	Development_FGF-family signaling	7.60E-02	FGF19, FGF4
**48**	Dysregulation of direct glucose-induced inhibition of Glucagon secretion from alpha-cells in type 2 diabetes	7.85E-02	CACNA1H, PAX6
**49**	Regulation of AKT(PKB)/ GSK3 beta cascade in bipolar disorder	8.11E-02	BDNF, GDNF
**50**	GTP metabolism	8.11E-02	Guanylate cyclase A (NPR1), PDE6C

Supplementary Table S4. Pathway analysis of dipeptidyl peptidase 7 (DPP7)-coexpressed genes from public breast cancer databases using the MetaCore database (with $p < 0.01$ set as the cut-off value).

#	**Maps**	***p*-Value**	**Network Objects from Active Data**
**1**	Cell cycle_Role of SCF complex in cell cycle regulation	1.77E-05	Skp2/TrCP/FBXW, Chk1, Cyclin D1, beta-TrCP, NEDD8, CKS1
**2**	DNA damage_ATM/ATR regulation of G1/S checkpoint	2.15E-05	PCNA, p38alpha (MAPK14), Cyclin A, Chk1, Cyclin D1, beta-TrCP, FBXW11
**3**	Role of histone modificators in progression of multiple myeloma	2.17E-05	EZH2, Cyclin D1, N-Ras, HDAC7, Tubulin alpha, HDAC2
**4**	Cell cycle_Role of APC in cell cycle regulation	3.21E-05	CDH1, Geminin, Cyclin A, Aurora-A, MAD2a, CKS1
**5**	Cell cycle_Spindle assembly and chromosome separation	3.86E-05	Importin (karyopherin)-alpha, TPX2, Aurora-A, NUMA1, Tubulin alpha, MAD2a
**6**	Cell cycle_Chromosome condensation in prometaphase	4.44E-05	CAP-C, Cyclin A, CAP-G/G2, Aurora-A, CAP-E
**7**	Cell cycle_Nucleocytoplasmic transport of CDK/Cyclins	1.25E-04	Importin (karyopherin)-alpha, Cyclin A, Cyclin D1, Cyclin D
**8**	Insulin-dependent stimulation of SREBP-1 in type 2 diabetes in liver	1.60E-04	SCAP, SREBP1 precursor, FASN, SREBP1 (Golgi membrane), SREBP1 (nuclear)
**9**	Cell cycle_Regulation of G1/S transition (part 2)	1.92E-04	Cyclin A2, p107, Cyclin A, Cyclin D1, Cyclin D
**10**	Transcription_Sirtuin6 regulation and functions	2.26E-04	SCAP, SREBP1 precursor, FASN, SREBP1 (Golgi membrane), USP10, SREBP1 (nuclear), LKB1
**11**	Histone deacetylases in Prostate Cancer	2.28E-04	HDAC11, HDAC9, HDAC7, Tubulin alpha, HDAC2
**12**	Abnormalities in cell cycle in SCLC	2.28E-04	PCNA, Cyclin A, Cyclin D1, Cyclin E2, CKS1
**13**	Development_Role of IL-8 in angiogenesis	2.75E-04	SCAP, SREBP1 precursor, FASN, SREBP1 (Golgi membrane), G-protein beta/gamma, EGFR, SREBP1 (nuclear)

**14**	Regulation of lipid metabolism_Regulation of fatty acid synthase activity in hepatocytes	4.48E-04	SREBP1 precursor, FASN, SREBP1 (Golgi membrane), SREBP1 (nuclear)
**15**	Protein folding_Membrane trafficking and signal transduction of G-alpha (i) heterotrimeric G-protein	4.48E-04	Adenylate cyclase type VI, G-protein beta, G-protein beta/gamma, G-protein gamma
**16**	DNA damage_ATM/ATR regulation of G2/M checkpoint: cytoplasmic signaling	4.67E-04	p38alpha (MAPK14), Chk1, Aurora-A, p38 MAPK, beta-TrCP, 14-3-3
**17**	Development_Positive regulation of STK3/4 (Hippo) pathway and negative regulation of YAP/TAZ function	4.76E-04	Skp2/TrCP/FBXW, TAZ, Adenylate cyclase, beta-TrCP, FasR(CD95), LKB1, 14-3-3
**18**	Cell cycle_ESR1 regulation of G1/S transition	5.66E-04	Cyclin A2, Cyclin A, Skp2/TrCP/FBXW, Cyclin D1, CKS1
**19**	Regulation of lipid metabolism_Regulation of lipid metabolism via LXR, NF-Y and SREBP	8.34E-04	SCAP, SREBP1 precursor, FASN, SREBP1 (Golgi membrane), SREBP1 (nuclear)
**20**	Cell cycle_Regulation of G1/S transition (part 1)	8.34E-04	Cyclin A, Skp2/TrCP/FBXW, Cyclin D1, beta-TrCP, Cyclin D
**21**	Transcription_Epigenetic regulation of gene expression	8.54E-04	Tip60, HDAC9, DNMT1, EZH2, Histone H2A, HDAC2
**22**	The role of UV radiation in melanoma development	1.06E-03	EGFR, p38 MAPK, N-Ras, XPD, FasR(CD95)
**23**	DNA damage_p53 activation by DNA damage	1.12E-03	p38alpha (MAPK14), Tip60, 14-3-3 theta, Chk1, p38 MAPK, 14-3-3
**24**	Development_Negative regulation of WNT/Beta-catenin signaling in the cytoplasm	1.13E-03	RACK1, G-protein beta/gamma, Skp2/TrCP/FBXW, TAZ, Cyclin D1, beta-TrCP, YAP1/TAZ
**25**	Oxidative stress_ROS-induced cellular signaling	1.30E-03	p38alpha (MAPK14), FASN, NRF2, Cyclin D1, p38 MAPK, HIF-prolyl hydroxylase, SREBP1 (nuclear), LKB1
**26**	HBV-dependent transcription regulation leading to HCC	1.33E-03	PCNA, SREBP1 precursor, FASN, Cyclin D1
**27**	SREBP1 cross-talk with PXR, CAR and LXR	1.55E-03	SCAP, SREBP1 precursor, SREBP1 (Golgi membrane), SREBP1 (nuclear)
**28**	DNA damage_ATM/ATR regulation of G2/M checkpoint: nuclear signaling	1.82E-03	CDH1, Cyclin A, DNMT1, Chk1, ATRIP
**29**	SCAP/SREBP Transcriptional Control of Cholesterol and FA Biosynthesis	1.82E-03	SCAP, SREBP1 precursor, FASN, SREBP1 (Golgi membrane), SREBP1 (nuclear)
**30**	Adiponectin in pathogenesis of type 2 diabetes	2.35E-03	SREBP1 precursor, p38alpha (MAPK14), FASN, SREBP1 (nuclear)

**31**	Main genetic and epigenetic alterations in lung cancer	2.43E-03	DNMT1, Cyclin D1, EGFR, IBP, LKB1
**32**	Transcription_Ligand-dependent activation of the ESR1/SP pathway	2.68E-03	TYSY, Cyclin D1, EGFR, Cyclin E2
**33**	Immune response_Function of MEF2 in T lymphocytes	2.91E-03	p38alpha (MAPK14), HDAC9, HDAC7, HDAC2, 14-3-3
**34**	SREBP1 cross-talk with PXR, CAR and LXR/ Rodent version	3.03E-03	SCAP, SREBP1 precursor, SREBP1 (Golgi membrane), SREBP1 (nuclear)
**35**	Cell cycle_Role of Nek in cell cycle regulation	3.40E-03	TPX2, Aurora-A, Tubulin alpha, MAD2a
**36**	DNA damage_Mismatch repair	3.40E-03	PCNA, DNMT1, USP10, MTMR15
**37**	Cytoskeleton remodeling_Substance P mediated membrane blebbing	3.48E-03	Substance P receptor, Tubulin alpha, MRLC
**38**	Development_EGFR signaling via small GTPases	3.82E-03	EGFR, N-Ras, RNTRE, E3b1(ABI-1)
**39**	Development_Differentiation of white adipocytes	4.08E-03	SREBP1 precursor, p38alpha (MAPK14), p107, PSAT, SREBP1 (nuclear)
**40**	G-protein signaling_Rap2A regulation pathway	4.17E-03	RAP-2A, M-Ras, cAMP-GEFI
**41**	MAPK-mediated proliferation of normal and asthmatic smooth muscle cells	4.77E-03	p38alpha (MAPK14), G-protein beta/gamma, Cyclin D1, EGFR, N-Ras
**42**	Role of FSH and Lutropin in ovarian cancer	4.77E-03	NRF2, Cyclin D1, EGFR, Adenylate cyclase, cAMP-GEFI
**43**	Transport_RAN regulation pathway	4.93E-03	NUP54, Importin (karyopherin)-alpha, NUP153
**44**	WNT signaling in gastric cancer	5.25E-03	Skp2/TrCP/FBXW, Cyclin D1, beta-TrCP, FBXW11
**45**	Signal transduction_IGF-1 receptor signaling pathway	5.54E-03	RACK1, MNK2(GPRK7), FASN, SREBP1 (nuclear), Cyclin D
**46**	Putative role of Estrogen receptor and Androgen receptor signaling in progression of lung cancer	5.54E-03	Cyclin D1, EGFR, p38 MAPK, SRD5A1, 14-3-3
**47**	Immune response_ETV3 affect on CSF1-promoted macrophage differentiation	5.77E-03	p38 MAPK, PRIM2A, HDAC2
**48**	Ethanol/Acetaldehyde-dependent stimulation of MMP-9 expression in HCC	5.77E-03	Skp2/TrCP/FBXW, p38 MAPK, beta-TrCP
**49**	G-protein signaling_G-Protein alpha-12 signaling pathway	6.38E-03	G-protein beta/gamma, TC21, M-Ras, p38 MAPK
**50**	Signal transduction_Cyclic AMP signaling	6.38E-03	Adenylate cyclase type VI, G-protein beta/gamma, RAP-2A, cAMP-GEFI

Supplementary Table S5. Pathway analysis of dipeptidyl peptidase 8 (DPP8)-coexpressed genes from public breast cancer databases using the MetaCore database (with $p < 0.01$ set as the cut-off value).

#	Maps	*p*-Value	Network Objects from Active Data
**1**	Development_Negative regulation of WNT/Beta-catenin signaling in the cytoplasm	5.91E-06	CXXC5, WWP1, TAZ, Cyclin D1, Casein kinase I alpha, Frizzled, YAP1/TAZ, Beclin 1, CDK6
**2**	Mitogenic action of ESR1 (membrane) in breast cancer	8.57E-06	ESR1 (nuclear), ESR1 (membrane), IGF-1 receptor, Chk1, Cyclin D1, EGFR, ErbB3
**3**	Main genetic and epigenetic alterations in lung cancer	9.90E-06	GSTP1, IBP4, IGF-1 receptor, Cyclin D1, EGFR, IBP, Bcl-2
**4**	IL-6 signaling in breast cancer cells	1.94E-05	ESR1 (nuclear), C/EBPbeta, gp130, Cyclin D1, Bcl-2, Fascin, IP10
**5**	Development_YAP/TAZ-mediated co-regulation of transcription	2.80E-05	SOD2, GATA-3, TAZ, Cyclin D1, TEF-3, SMAD3, CDK6
**6**	Immune response_IL-4-induced regulators of cell growth, survival, differentiation and metabolism	5.48E-05	PLEKHF1, GATA-3, CISH, Bcl-2, Cyclin D, SOCS1, CDK6
**7**	G-protein signaling_Regulation of Cyclic AMP levels by ACM	7.34E-05	Adenylate cyclase type VI, G-protein alpha-q, G-protein alpha-i family, G-protein alpha-i3, G-protein alpha-i2, G-protein alpha-q/11
**8**	Role of DNA methylation in progression of multiple myeloma	7.34E-05	ESR1 (nuclear), SOD2, Cyclin D1, Frizzled, SOCS1, CDK6
**9**	wtCFTR and deltaF508 traffic / Membrane expression (normal and CF)	9.43E-05	Rabaptin-5, Keratin 18, EBP50, Filamin B (TABP), Calreticulin, Rab-11A
**10**	Signal transduction_Adenosine A3 receptor signaling pathway	1.06E-04	G-protein alpha-i family, G-protein alpha-i3, G-protein alpha-i2, Adenylate cyclase, Bcl-2, G-protein alpha-q/11
**11**	Deregulation of canonical WNT signaling in major depressive disorder	1.18E-04	Casein kinase I gamma 1, Cyclin D1, Tau (MAPT), FZD9, Frizzled
**12**	Immune response_IFN-alpha/beta signaling via MAPKs	1.57E-04	IFNAR1, Filamin B (TABP), Cyclin D1, PIAS1, SMAD3, MEK4(MAP2K4), IP10
**13**	Regulation of AKT(PKB)/ GSK3 beta cascade in bipolar disorder	2.07E-04	ESR1 (membrane), IGF-1 receptor, G-protein alpha-i family, G-protein alpha-i3, G-protein alpha-i2, GFRalpha1
**14**	PR action in breast cancer: stimulation of cell growth and proliferation	2.46E-04	ESR1 (nuclear), ESR1 (membrane), Cyclin D1, EGFR, ErbB3
**15**	Apoptosis and survival_Endoplasmic reticulum stress response pathway	2.53E-04	SOD2, XBP1, tBid, Bcl-2, MEK4(MAP2K4), Bid

**16**	Development_Negative feedback regulation of WNT/Beta-catenin signaling	2.80E-04	CXXC5, G-protein alpha-q, Cyclin D1, G-protein alpha-i family, Frizzled
**17**	Putative role of Estrogen receptor and Androgen receptor signaling in progression of lung cancer	3.07E-04	ESR1 (nuclear), ESR1 (membrane), Cyclin D1, EGFR, G-protein alpha-i family, Bcl-2
**18**	Cytoskeleton remodeling_ACM3 and ACM4 in keratinocyte migration	3.19E-04	Adenylate cyclase type VI, G-protein alpha-q, G-protein alpha-i family, G-protein alpha-i2, G-protein alpha-q/11
**19**	Amitraz-induced inhibition of Insulin secretion	3.64E-04	Adenylate cyclase type VI, G-protein alpha-i family, G-protein alpha-i3, G-protein alpha-i2
**20**	Regulation of CFTR activity (normal and CF)	4.43E-04	EBP50, Filamin B (TABP), AMPK beta subunit, G-protein alpha-i family, Adenylate cyclase, Calcineurin A (catalytic)
**21**	Regulation of metabolism_Bile acids regulation of glucose and lipid metabolism via FXR	4.58E-04	SREBP1 precursor, HNF3-alpha, SREBP1 (nuclear), HNF3, F16P
**22**	Apoptosis and survival_BAD phosphorylation	5.13E-04	IGF-1 receptor, EGFR, Calcineurin A (catalytic), Bcl-2, Beclin 1
**23**	Transport_ACM3 signaling in salivary glands	5.13E-04	Adenylate cyclase type VI, G-protein alpha-q, G-protein alpha-i family, G-protein alpha-i3, G-protein alpha-q/11
**24**	NF-AT signaling in cardiac hypertrophy	5.72E-04	IGF-1 receptor, gp130, G-protein alpha-i family, Calcineurin A (catalytic), MAP2K5 (MEK5), G-protein alpha-q/11
**25**	Development_WNT/Beta-catenin signaling pathway. Signalosome	5.73E-04	Casein kinase I gamma 1, G-protein alpha-q, G-protein alpha-i family, Casein kinase I alpha, Frizzled
**26**	Apoptosis and survival_TNFR1 signaling pathway	5.73E-04	jBid, tBid, Bcl-2, MEK4(MAP2K4), Bid
**27**	Development_Estrogen-independent activation of ESR1 and ESR2	6.39E-04	ESR1 (nuclear), IGF-1 receptor, Cyclin D1, EGFR, ErbB3
**28**	Ligand-independent activation of Androgen receptor in Prostate Cancer	6.73E-04	IGF-1 receptor, Cyclin D1, EGFR, Frizzled, FRS2, ErbB3
**29**	Role of neuropeptides in pathogenesis of SCLC	6.73E-04	AVP extracellular region, G-protein alpha-q, EGFR, Galanin, G-protein alpha-q/11, ErbB3
**30**	Immune response_IL-3 signaling via JAK/STAT, p38, JNK and NF-kB	6.97E-04	C/EBPbeta, XBP1, Cyclin D1, CISH, Bcl-2, MEK4(MAP2K4), SOCS1
**31**	Development_Membrane-bound ESR1: interaction with growth factors signaling	7.09E-04	ESR1 (nuclear), ESR1 (membrane), IGF-1 receptor, Cyclin D1, ErbB3

**32**	Transport_Alpha-2 adrenergic receptor regulation of ion channels	8.68E-04	Adenylate cyclase type VI, G-protein alpha-q, G-protein alpha-i family, G-protein alpha-i3, G-protein alpha-i2
**33**	Immune response_IL-6 signaling pathway via JAK/STAT	9.17E-04	C/EBPbeta, gp130, CISH, MEK4(MAP2K4), SOCS1, CDK6
**34**	Neurophysiological process_ACM regulation of nerve impulse	9.56E-04	G-protein alpha-q, G-protein alpha-i family, G-protein alpha-i2, Calcineurin A (catalytic), G-protein alpha-q/11
**35**	Neurophysiological process_Thyroliberin signaling	1.06E-03	G-protein alpha-q, G-protein alpha-i3, G-protein alpha-i2, Adenylate cyclase, Tau (MAPT), G-protein alpha-q/11
**36**	Cell cycle_Role of SCF complex in cell cycle regulation	1.08E-03	SKP1, Chk1, Cyclin D1, SMAD3
**37**	Development_Thromboxane A2 signaling pathway	1.15E-03	G-protein alpha-q, Cyclin D1, EGFR, G-protein alpha-i family, Adenylate cyclase
**38**	PI3K signaling in gastric cancer	1.15E-03	G-protein alpha-q, Cyclin D1, EGFR, G-protein alpha-q/11, ErbB3
**39**	Protein folding and maturation_Posttranslational processing of neuroendocrine peptides	1.15E-03	AVP extracellular region, AVP-NPII, AVP-Gly, AVP-Gly-Lys-Arg, Neurophysin-II
**40**	EGFR family signaling in pancreatic cancer	1.22E-03	BFL1, Cyclin D1, EGFR, Bcl-2, MEK4(MAP2K4), ErbB3
**41**	DNA damage_Brca1 as a transcription regulator	1.24E-03	ESR1 (nuclear), IGF-1 receptor, XPC, Cyclin D1
**42**	Transcription_Ligand-dependent activation of the ESR1/SP pathway	1.24E-03	ESR1 (nuclear), C/EBPbeta, Cyclin D1, EGFR
**43**	Colorectal cancer (general schema)	1.24E-03	IGF-1 receptor, gp130, EGFR, Frizzled
**44**	Putative pathways of n-3 and n-6 fatty acids in obesity and type 2 diabetes	1.26E-03	SREBP1 precursor, C/EBPbeta, Adenylate cyclase, SREBP1 (nuclear), G-protein alpha-q/11
**45**	Development_Endothelin-1/EDNRA signaling	1.38E-03	Cyclin D1, G-protein alpha-i family, Adenylate cyclase, MEK4(MAP2K4), G-protein alpha-q/11
**46**	Signal transduction_Angiotensin II/ AGTR1 signaling via TGF-beta 1 and SMADs	1.38E-03	SREBP1 precursor, SREBP1 (nuclear), SMAD3, MEK4(MAP2K4), NALP3
**47**	Role of inhibition of WNT signaling in the progression of lung cancer	1.40E-03	Keratin 18, FZD9, Frizzled, MAP2K5 (MEK5)
**48**	DNA damage_ATR activation by DNA damage	1.50E-03	Chk1, RAD17, ATRIP, p53BP1, Bid
**49**	EGFR signaling pathway in lung cancer	1.50E-03	gp130, Cyclin D1, EGFR, Bcl-2, ErbB3

**50**	Apoptosis and survival_Regulation of apoptosis by mitochondrial proteins	1.51E-03	BFL1, tBid, Calcineurin A (catalytic), Bcl-2, Beclin 1, MAP1, Bid

Supplementary Table S6. Pathway analysis of dipeptidyl peptidase 9 (DPP9)-coexpressed genes from public breast cancer databases using the MetaCore database (with $p < 0.01$ set as the cut-off value).

#	**Maps**	***p*-Value**	**Network Objects from Active Data**
**1**	Immune response_IFN-alpha/beta signaling via PI3K and NF-kB pathways	2.03E-08	NF-kB2 (p100), AKT1, NF-kB2 (p52), MNK2(GPRK7), Tyk2, NF-kB, MEK1/2, Tuberin, TRAF2, AKT(PKB), PI3K reg class IA (p85)
**2**	Immune response_TNF-R2 signaling pathways	6.57E-08	NF-kB2 (p100), Bcl-XL, NF-kB2 (p52), NF-kB, TRAF2, AKT(PKB), PI3K reg class IA (p85), PI3K reg class IA
**3**	Development_GM-CSF signaling	2.44E-06	CISH, Bcl-XL, NF-kB, MEK2(MAP2K2), PI3K reg class IA (p85-beta), AKT(PKB), PI3K reg class IA (p85)
**4**	Main growth factor signaling cascades in multiple myeloma cells	1.01E-05	NF-kB, MEK2(MAP2K2), PI3K reg class IA (p85-beta), AKT(PKB), PI3K reg class IA (p85), PI3K reg class IA
**5**	Apoptosis and survival_IL-17-induced CIKS-independent signaling pathways	2.00E-05	AKT1, Tyk2, NF-kB, MEK2(MAP2K2), PI3K reg class IA (p85), PI3K reg class IA
**6**	Immune response_OX40L/ OX40 signaling pathway	2.16E-05	NF-kB2 (p100), Bcl-XL, NF-kB2 (p52), NF-kB, TRAF2, AKT(PKB), PI3K reg class IA (p85)
**7**	Survival pathways in Prostate Cancer	3.25E-05	Bcl-XL, NF-kB, Tuberin, MEK2(MAP2K2), AKT(PKB), PI3K reg class IA
**8**	Immune response_IL-23 signaling pathway	3.52E-05	AKT1, Tyk2, NF-kB, AKT(PKB), PI3K reg class IA
**9**	Immune response_BAFF-induced signaling	3.65E-05	AKT1, MEK1/2, Tuberin, TRAF2, AKT(PKB), PI3K reg class IA (p85)
**10**	Some pathways of EMT in cancer cells	3.65E-05	Axin, PDGF-B, Tuberin, TRAF2, AKT(PKB), PI3K reg class IA
**11**	G-protein signaling_Proinsulin C-peptide signaling	4.09E-05	NF-kB, TRAF2, MEK2(MAP2K2), AKT(PKB), PI3K reg class IA (p85), PI3K reg class IA
**12**	IL-2 as a growth factor for T cells in multiple sclerosis	4.82E-05	Bcl-XL, NF-kB, Tuberin, AKT(PKB), PI3K reg class IA (p85)
**13**	Role of IFN-beta in inhibition of Th1 cell differentiation in multiple sclerosis	5.59E-05	SHP-1, NF-kB, AKT(PKB), PI3K reg class IA (p85), PI3K reg class IA
**14**	NF-kB pathway in multiple myeloma	6.46E-05	NF-kB2 (p100), Bcl-XL, NF-kB2 (p52), NF-kB, TRAF2
**15**	Signal transduction_IGF-1 receptor signaling pathway	7.64E-05	Bcl-XL, SREBP1 (nuclear), MNK2(GPRK7), MEK1/2, AKT(PKB), PI3K reg class IA (p85)
**16**	Oxidative stress_Activation of NADPH oxidase	8.42E-05	p114-RhoGEF, AKT1, MEK1/2, PREX1, AKT(PKB), PI3K reg class IA
**17**	IGF family signaling in colorectal cancer	9.26E-05	GIPC, Bcl-XL, MNK2(GPRK7), NF-kB, MEK1/2, AKT(PKB)
**18**	Immune response_TSLP signaling	1.10E-04	CISH, NF-kB, MEK1/2, AKT(PKB), PI3K reg class IA
**19**	Immune response_IL-10 signaling pathway	1.11E-04	CRP2, Bcl-XL, Tyk2, NF-kB, AKT(PKB), PI3K reg class IA

**20**	Immune response_IL-18 signaling	1.22E-04	CD147, NF-kB, MEK1/2, Bcl-XS, AKT(PKB), PI3K reg class IA
**21**	MAPK-independent proliferation of normal and asthmatic smooth muscle cells	1.33E-04	PDGF-B, NF-kB, AKT(PKB), PI3K reg class IA (p85), PI3K reg class IA, G-protein alpha-q/11
**22**	Development_Neurotrophin family signaling	1.40E-04	SH2B, MEK2(MAP2K2), AKT(PKB), PI3K reg class IA (p85), PI3K reg class IA
**23**	Transcription_Sin3 and NuRD in transcription regulation	1.40E-04	ARID4B, Sin3B, MBD3, Mi-2 beta, Mi-2
**24**	Development_Role of IL-8 in angiogenesis	1.45E-04	SREBP1 precursor, SREBP1 (nuclear), NF-kB, SREBP1 (Golgi membrane), AKT(PKB), PI3K reg class IA (p85)
**25**	Apoptosis and survival_Anti-apoptotic TNFs/NF-kB/Bcl-2 pathway	1.58E-04	NF-kB2 (p100), Bcl-XL, NF-kB2 (p52), NF-kB, TRAF2
**26**	Development_Thrombopoietin signaling via JAK-STAT pathway	1.65E-04	CISH, Bcl-XL, Tyk2, Oct-1
**27**	HGF receptor (Met) and MSP receptor (RON) signaling pathways in SCLC	1.77E-04	AKT1, MEK1/2, Alpha adducin, AKT(PKB), PI3K reg class IA (p85)
**28**	Signal transduction_AKT signaling	1.77E-04	Bcl-XL, NF-kB, Tuberin, AKT(PKB), PI3K reg class IA
**29**	Apoptosis and survival_Anti-apoptotic action of Gastrin	1.77E-04	Bcl-XL, MEK2(MAP2K2), AKT(PKB), PI3K reg class IA (p85), G-protein alpha-q/11
**30**	Signal transduction_mTORC2 downstream signaling	1.87E-04	AKT1, PRAS40, SREBP1 (nuclear), Tuberin, PREX1, AKT(PKB)
**31**	Chemotaxis_Lysophosphatidic acid signaling via GPCRs	1.94E-04	AKT1, Bcl-XL, MEK1/2, PREX1, AKT(PKB), PI3K reg class IA (p85), PRK1, G-protein alpha-q/11
**32**	IGF signaling in lung cancer	1.98E-04	Bcl-XL, Tuberin, MEK2(MAP2K2), AKT(PKB), PI3K reg class IA
**33**	Stellate cells activation and liver fibrosis	2.19E-04	PDGF-B, SMAD4, TRAF2, MEK2(MAP2K2), AKT(PKB), PI3K reg class IA (p85)
**34**	Apoptosis and survival_Apoptotic Activin A signaling	2.31E-04	Bcl-XL, SMAD4, MEK2(MAP2K2), AKT(PKB)
**35**	Development_Stimulation of differentiation of mouse embryonic fibroblasts into adipocytes by extracellular factors	2.37E-04	AKT1, SREBP1 precursor, SMAD4, Tuberin, MEK2(MAP2K2), PI3K reg class IA (p85)
**36**	Regulation of GSK3 beta in bipolar disorder	2.45E-04	Axin, AKT1, MEK1/2, PP2A regulatory, AKT(PKB)
**37**	Development_Endothelin-1/EDNRA transactivation of EGFR	2.45E-04	Tuberin, MEK2(MAP2K2), AKT(PKB), PI3K reg class IA, G-protein alpha-q/11
**38**	Canonical Leptin pathways in breast cancer	2.71E-04	Axin, NF-kB, MEK1/2, AKT(PKB), PI3K reg class IA
**39**	Signal transduction_mTORC1 upstream signaling	2.97E-04	TELO2, Axin, PRAS40, MEK1/2, Tuberin, AKT(PKB)

**40**	EGFR family signaling in pancreatic cancer	3.20E-04	Bcl-XL, NF-kB, Tuberin, MEK2(MAP2K2), AKT(PKB), PI3K reg class IA (p85)
**41**	Activation of TNF-alpha-dependent pro-tumoral effect in colorectal cancer	3.30E-04	Axin, NF-kB, MADD, MEK1/2, TRAF2
**42**	Tissue Factor signaling in cancer via PAR1 and PAR2	3.30E-04	Bcl-XL, MEK2(MAP2K2), AKT(PKB), PI3K reg class IA, G-protein alpha-q/11
**43**	Development_G-CSF signaling	3.30E-04	Bcl-XL, Tyk2, MEK2(MAP2K2), AKT(PKB), PI3K reg class IA (p85)
**44**	IL-6 signaling in multiple myeloma	3.99E-04	Bcl-XL, Tyk2, MEK2(MAP2K2), AKT(PKB), PI3K reg class IA (p85)
**45**	Anti-apoptotic action of ErbB2 in breast cancer	3.99E-04	AKT1, MEK1/2, Tuberin, AKT(PKB), PI3K reg class IA (p85)
**46**	Immune response_B cell antigen receptor (BCR) pathway	4.19E-04	Bcl-XL, NF-kB, MEK1/2, PIP5KI, MEK2(MAP2K2), AKT(PKB), PI3K reg class IA (p85)
**47**	Development_Endothelin-1/EDNRA signaling	4.37E-04	MEK2(MAP2K2), FARP2, AKT(PKB), PI3K reg class IA, G-protein alpha-q/11
**48**	Signal transduction_Angiotensin II/ AGTR1 signaling via TGF-beta 1 and SMADs	4.37E-04	SREBP1 precursor, SREBP1 (nuclear), SMAD4, NF-kB, AKT(PKB)
**49**	Main pathways of Schwann cells transformation in neurofibromatosis type 1	4.54E-04	Bcl-XL, PDGF-B, MEK1/2, Tuberin, AKT(PKB), PI3K reg class IA (p85)
**50**	Immune response_BAFF-induced non-canonical NF-kB signaling	4.76E-04	NF-kB2 (p100), NF-kB2 (p52), UBC12, TRAF2

Supplementary Table S7. Pathway analysis of dipeptidyl peptidase 10 (DPP10)-coexpressed genes from public breast cancer databases using the MetaCore database (with $p < 0.01$ set as the cut-off value).

#	**Maps**	***p*-Value**	**Network Objects from Active Data**
**1**	Cell cycle_Role of APC in cell cycle regulation	7.96E-07	BUB1, CDC25A, SKP2, Tome-1, ORC1L, Aurora-A
**2**	Higher ESR1 / ESR2 ratio in breast cancer	3.20E-05	PTEN, CDC25A, SKP2, BTG2, FOXM1
**3**	Cell cycle_The metaphase checkpoint	3.20E-05	BUB1, HEC, CENP-F, Survivin, Aurora-A
**4**	Putative pathways of hormone action in neurofibromatosis type 1	1.17E-04	PI3K reg class IA (p85-alpha), PMP22, PI3K reg class IA (p85), PI3K reg class IA
**5**	Nicotine / nAChR alpha-3/nAChR beta-2 signaling in NSCLC	1.37E-04	CDC25A, PI3K reg class IA (p85-alpha), E2F2, Survivin
**6**	Survival pathways in Prostate Cancer	1.60E-04	PTEN, TRID(TNFRSF10C), Apo-2L(TNFSF10), Survivin, PI3K reg class IA
**7**	Cell cycle_Role of Nek in cell cycle regulation	3.15E-04	HEC, TPX2, Aurora-A, PI3K reg class IA
**8**	Degranulation of lung mast cells	4.78E-04	Neurokinin A, Urocortin, Substance P extracellular region, PGE2R3, PI3K reg class IA (p85)
**9**	Resistance of melanoma cells to Apo-2L(TNFSF10)-induced apoptosis	6.81E-04	TRID(TNFRSF10C), Apo-2L(TNFSF10), Survivin, PI3K reg class IA (p85)

**10**	Main growth factor signaling cascades in multiple myeloma cells	8.25E-04	PTEN, PI3K reg class IA (p85-alpha), PI3K reg class IA (p85), PI3K reg class IA
**11**	PI3K signaling in gastric cancer	1.75E-03	PTEN, PI3K reg class IA (p85-alpha), PI3K reg class IA (p85), PI3K reg class IA
**12**	IL-6 signaling in breast cancer cells	2.17E-03	Survivin, IP10, gp130, PI3K reg class IA
**13**	Immune response_Inhibitory PD-1 signaling in T cells	2.17E-03	PTEN, SKP2, PI3K reg class IA (p85), Skp2/TrCP/FBXW
**14**	Development_Cytokine-mediated regulation of megakaryopoiesis	2.83E-03	PI3K reg class IA (p85), gp130, PI3K reg class IA, MYCT1
**15**	Cell cycle_Role of SCF complex in cell cycle regulation	3.25E-03	CDC25A, SKP2, Skp2/TrCP/FBXW
**16**	IGF family signaling in colorectal cancer	3.42E-03	PTEN, PI3K reg class IA (p85-alpha), MAFG, Apo-2L(TNFSF10)
**17**	Role of IFN-beta in activation of T cell apoptosis in multiple sclerosis	3.59E-03	TRID(TNFRSF10C), Apo-2L(TNFSF10), Survivin
**18**	Macrophage and dendritic cell phenotype shift in cancer	3.78E-03	c-Rel (NF-kB subunit), Apo-2L(TNFSF10), MSR1, IP10, PI3K reg class IA
**19**	Immune response_IL-4-induced regulators of cell growth, survival, differentiation and metabolism	3.85E-03	CDC25A, MCM4, A-FABP, DHA2
**20**	Role of IL-2 in the enhancement of NK cell cytotoxicity in multiple sclerosis	3.94E-03	CX3CR1, Apo-2L(TNFSF10), PI3K reg class IA (p85)
**21**	MAPK-independent proliferation of normal and asthmatic smooth muscle cells	4.31E-03	PI3K reg class IA (p85-alpha), PLAT (TPA), PI3K reg class IA (p85), PI3K reg class IA
**22**	Cell cycle_Start of DNA replication in early S phase	4.32E-03	MCM10, MCM4, ORC1L
**23**	Resistance of pancreatic cancer cells to death receptor signaling	4.71E-03	TRID(TNFRSF10C), Apo-2L(TNFSF10), Survivin
**24**	Cell cycle_Spindle assembly and chromosome separation	4.71E-03	HEC, TPX2, Aurora-A
**25**	Immune response_IL-11 signaling via JAK/STAT	5.13E-03	A-FABP, Survivin, gp130
**26**	Role of Apo-2L(TNFSF10) in Prostate Cancer cell apoptosis	5.13E-03	TRID(TNFRSF10C), Apo-2L(TNFSF10), Survivin
**27**	Development_Angiopoietin - Tie2 signaling	5.57E-03	Survivin, PI3K reg class IA, TIE2
**28**	IL-6 signaling pathway in lung cancer	5.57E-03	Survivin, gp130, PI3K reg class IA
**29**	Cell cycle_ESR1 regulation of G1/S transition	5.57E-03	CDC25A, SKP2, Skp2/TrCP/FBXW
**30**	CHDI_Correlations from Discovery data_Causal network (positive)	6.03E-03	PI3K reg class IA (p85), gp130, TIE2
**31**	Blood coagulation_Blood coagulation	7.55E-03	Alpha 1-antitrypsin, PLAT (TPA), Protein C
**32**	Th2-cytokines-induced mucous metaplasia in asthma	8.10E-03	SOX17, PGE2R3, PI3K reg class IA
**33**	Development_FGF signaling in pancreatic and hepatic differentiation of embryonic stem cells	8.10E-03	Alpha 1-antitrypsin, Albumin, SOX17
**34**	IL-6 signaling in Prostate Cancer	8.10E-03	PTEN, gp130, PI3K reg class IA

**35**	Chemoresistance pathways mediated by constitutive activation of PI3K pathway and BCL-2 in small cell lung cancer	9.27E-03	Survivin, PI3K reg class IA (p85), PI3K reg class IA
**36**	Development_TGF-beta family mediated differentiation of embryonic stem cells	9.27E-03	SOX17, CD34, Transthyretin
**37**	Regulation of immune cell differentiation by Notch signaling	9.89E-03	PI3K reg class IA (p85-alpha), IFI16, PI3K reg class IA (p85)
**38**	Leptin signaling in colorectal cancer	1.05E-02	PTEN, Survivin, PI3K reg class IA
**39**	Mechanisms of resistance to EGFR inhibitors in lung cancer	1.12E-02	PTEN, Survivin, PI3K reg class IA (p85)
**40**	Development_Schema: FGF signaling in embryonic stem cell self-renewal and differentiation	1.26E-02	Albumin, SOX17, CD34
**41**	K-RAS signaling in lung cancer	1.34E-02	PTEN, BTG2, PI3K reg class IA (p85)
**42**	IGF-1 signaling in multiple myeloma	1.49E-02	PTEN, Survivin, PI3K reg class IA
**43**	The role of PTEN and PI3K signaling in melanoma	1.49E-02	PTEN, Survivin, PI3K reg class IA (p85)
**44**	HBV-dependent NF-kB and PI3K/AKT pathways leading to HCC	1.49E-02	PTEN, PI3K reg class IA (p85-alpha), PI3K reg class IA (p85)
**45**	Development_Signaling pathways in embryonic hepatocyte maturation	1.57E-02	APOA2, gp130, PI3K reg class IA
**46**	Development_Endothelial differentiation during embryonic development	1.57E-02	Survivin, PI3K reg class IA, TIE2
**47**	Neurophysiological process_GABAergic neurotransmission	1.57E-02	GLNA, GABT, GBR2
**48**	DNA damage_ATM/ATR regulation of G2/M checkpoint: cytoplasmic signaling	1.57E-02	UBE2C, CDC25A, Aurora-A
**49**	G-protein signaling_Proinsulin C-peptide signaling	1.66E-02	PI3K reg class IA (p85-alpha), PI3K reg class IA (p85), PI3K reg class IA
**50**	Regulation of VEGF expression in lung cancer	1.74E-02	PI3K reg class IA (p85), gp130, PI3K reg class IA
